# Designing and engineering evolutionary robust genetic circuits

**DOI:** 10.1186/1754-1611-4-12

**Published:** 2010-11-01

**Authors:** Sean C Sleight, Bryan A Bartley, Jane A Lieviant, Herbert M Sauro

**Affiliations:** 1 Department of Bioengineering, University of Washington, Seattle, WA 98195, USA

## Abstract

**Background:**

One problem with engineered genetic circuits in synthetic microbes is their stability over evolutionary time in the absence of selective pressure. Since design of a selective environment for maintaining function of a circuit will be unique to every circuit, general design principles are needed for engineering evolutionary robust circuits that permit the long-term study or applied use of synthetic circuits.

**Results:**

We first measured the stability of two BioBrick-assembled genetic circuits propagated in *Escherichia coli *over multiple generations and the mutations that caused their loss-of-function. The first circuit, T9002, loses function in less than 20 generations and the mutation that repeatedly causes its loss-of-function is a deletion between two homologous transcriptional terminators. To measure the effect between transcriptional terminator homology levels and evolutionary stability, we re-engineered six versions of T9002 with a different transcriptional terminator at the end of the circuit. When there is no homology between terminators, the evolutionary half-life of this circuit is significantly improved over 2-fold and is independent of the expression level. Removing homology between terminators and decreasing expression level 4-fold increases the evolutionary half-life over 17-fold. The second circuit, I7101, loses function in less than 50 generations due to a deletion between repeated operator sequences in the promoter. This circuit was re-engineered with different promoters from a promoter library and using a kanamycin resistance gene (*kanR*) within the circuit to put a selective pressure on the promoter. The evolutionary stability dynamics and loss-of-function mutations in all these circuits are described. We also found that on average, evolutionary half-life exponentially decreases with increasing expression levels.

**Conclusions:**

A wide variety of loss-of-function mutations are observed in BioBrick-assembled genetic circuits including point mutations, small insertions and deletions, large deletions, and insertion sequence (IS) element insertions that often occur in the scar sequence between parts. Promoter mutations are selected for more than any other biological part. Genetic circuits can be re-engineered to be more evolutionary robust with a few simple design principles: high expression of genetic circuits comes with the cost of low evolutionary stability, avoid repeated sequences, and the use of inducible promoters increases stability. Inclusion of an antibiotic resistance gene within the circuit does not ensure evolutionary stability.

## Background

Synthetic biology is the design and engineering of new biological functions and systems that do not occur in nature. This relatively new field has provided insight into the mechanisms of natural gene networks [1,2] and engineered multicellular pattern formations [3], bacterial photography [4], tumor-targeting bacteria [5], feed-forward network based concentration sensors [6], robust and tunable oscillators [7], and genetic networks that count [8]. On the genome level, entire metabolic pathways have been engineered to overproduce an anti-malaria compound [9], biofuels from plant biomass [10,11] lycopene through automated genome engineering and accelerated evolution [12], and a synthetic chromosome [13] transplanted into a host bacterium [14]. Despite recent efforts of engineering at the genome level, most synthetic biology constructs are engineered at the level of genetic circuits encoded on plasmids.

Genetic circuits are built bottom-up from biological parts. A biological part is a DNA sequence that encodes a basic biological function [15]. Examples of parts include promoters, ribosome binding sites (RBS), protein or RNA coding regions, and transcriptional terminators. Biological engineers can assemble individual parts or combination of parts together using a BioBrick assembly standard for physical composition [16] (described in [17]). Parts that conform to this BioBrick assembly standard are BioBrick standard biological parts, or BioBricks. Standard Assembly involves digestion of two BioBricks encoded on plasmids with different restriction enzymes that leave compatible sticky ends which can be ligated together into a new BioBrick. This assembly method effectively replaces the restriction sites between the assembled parts with a 'scar' sequence, allowing for the new BioBrick to be later combined with other BioBricks. Alternative assembly strategies have recently been proposed [18,19] to improve upon the original assembly standard. The MIT Registry of Standard Biological Parts (called "The Registry" from here on) maintains over 3000 BioBricks encoded on plasmids that are available to researchers with a wide variety of different functions, from bacterial photography, to quorum sensing to odor production and sensing.

BioBricks are widely available for the design of more complex systems, but in general are not well-characterized [15,17]. The most well-characterized part to date is a cell-cell communication receiver device [17], which was provided with a published prototype "biological part datasheet" containing information engineers would need to use it in their own designs. One of the figures in this datasheet describes the reliability of this circuit over evolutionary time. Connecting the receiver device to a GFP-reporter device causes this circuit to repeatedly lose function in less than 100 generations due to a deletion mutation between transcriptional terminators that are repeated in both the receiver and reporter devices. Another example of genetic circuits losing function over evolutionary time is illustrated by studies of microchemostat-evolved strains containing a cell density regulation circuit that loses function in less than 100 hours [20,21]. The evolutionary stability of whole circuits is therefore an emergent property of the context of its biological parts.

Evolutionary stability is a problem in genetic circuits if there is no selective pressure to maintain function of the circuit. The current belief is that this loss-of-function occurs because any cell in the population that acquires a mutation in the genetic circuit often has a growth advantage and can outcompete the cells in the population with all functional plasmids. As the cells divide, any cell with a larger percentage of mutant plasmids will eventually dominate the population until only cells with mutant plasmids remain. A simulation study predicted that the time for a non-functional mutant of a synthetic microbe to become the majority of the population is a function of the growth rate difference between the mutant and functional cells, circuit size, circuit architecture, and mutation rate [22]. Non-functional mutants often have a growth advantage because a mutation that inactivates a genetic circuit can reduce its metabolic load. The magnitude of metabolic load caused by expression and replication of foreign genes is dependent on many factors such as plasmid size, plasmid copy number, the foreign gene being expressed, antibiotic resistance gene, metabolic state of the cell, growth media, and amount of dissolved oxygen in the media [23]. Dekel and Alon [24] directly measured the cost associated with expression and maintenance of Lac proteins when they provided no fitness benefit and found mutations that alleviated this cost in the non-selective environment. There are also examples of chromosomal genes that have lost function over evolutionary time when not under selection [25,26] and so encoding synthetic circuits into the chromosome will only delay this problem.

The evolutionary stability of genetic circuits within synthetic microbes will be an increasingly significant issue as these circuits become more complex and need to be functional over longer periods of time. The ability to engineer evolutionary robust genetic circuits will be important for applied uses of synthetic microbes that perform long-term functions in the environment and possibly in the human body. This ability will also be important for the study of genetic circuits in microchemostats and microfluidic devices over multiple generations. Ideally, a selective regime should be used to maintain circuit function over evolutionary time. However, design of a selective regime for synthetic microbes is unique to the genetic circuit of interest, and design for maintaining function of a particular circuit is often difficult. Therefore, general design principles are needed for engineering evolutionary robust circuits that will maximize stability over time.

As a first step towards this goal, this study aimed to understand the loss-of-function mutations that occur in two genetic circuits over evolutionary time and their evolutionary stability dynamics. Next, we re-engineered these circuits in various ways to determine the predictability of mutations in replicate evolved populations and whether we could make these circuits more evolutionary robust. The results from these experiments allowed us to observe the mutations in several diverse circuits, determine their evolutionary stability dynamics, and develop simple design principles for engineering evolutionary robust circuits.

## Results

### Loss-of-function mutations and evolutionary stability dynamics in two genetic circuits

We first measured the evolutionary stability dynamics of two genetic circuits propagated in *Escherichia coli *MG1655 in order to determine the loss-of-function mutations that cause their instability and which circuit is the most robust over evolutionary time. High-copy plasmids were used instead of low or medium-copy plasmids to maximize selective pressure so that evolution would occur more rapidly since replication and expression of genetic circuits encoded on high-copy plasmids will increase metabolic load and lower fitness. Cells with a low metabolic load (e.g., cells with mutant plasmids) have greater fitness than cells with a higher metabolic load (e.g., cells with functional plasmids) (unpublished results). Therefore, we expect that mutants will be able to rapidly outcompete functional cells that have a high expression level. However, other factors besides expression level will play a role in this evolutionary process such as mutation rate and the metabolic load associated with plasmid replication.

**Figure 1:**
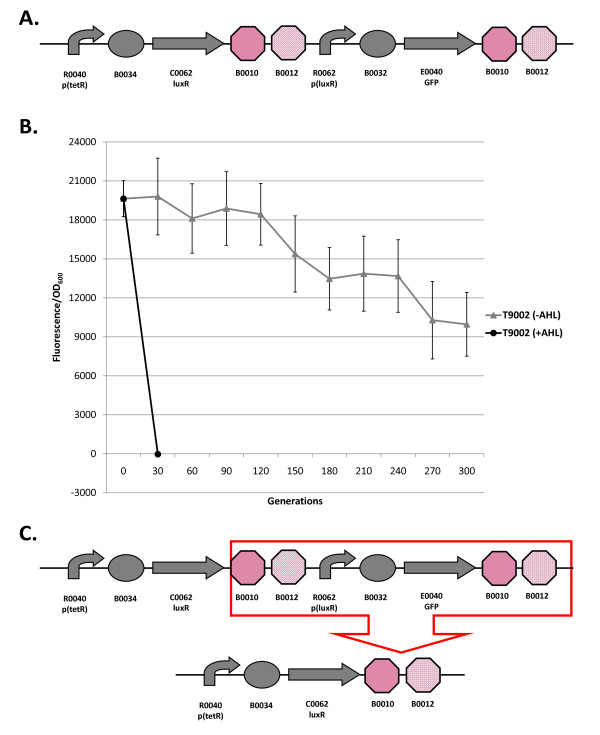
**Loss-of-function mutations and evolutionary stability dynamics in T9002**. (A) The T9002 genetic circuit. Symbols depict promoters (bent arrows), ribosome binding sites (ovals), coding sequences (arrows), and transcriptional terminators (octagons). T9002 consists of two devices, a luxR receiver device and a GFP-expressing device. The first device is composed of the *tetR*-regulated promoter R0040 that is constitutively expressed in the MG1655 strain since it does not produce TetR, B0034 RBS, C0062 luxR coding sequence, and B0010-B0012 (B0015) transcriptional terminator. The second device is composed of the R0062 luxR promoter, B0032 RBS, E0040 GFP coding sequence, and B0015 transcriptional terminator. LuxR is constitutively expressed from the *tetR *promoter. When the inducer 3OC_6_HSL (AHL) is added to the media, it binds with LuxR to activate transcription of GFP from the *luxR *promoter. If no AHL is in the media, the circuit is off. (B) Evolutionary stability dynamics of T9002 evolved under low (-AHL) and high (+AHL) input conditions. Low and high input evolved populations are shown with solid gray triangles and solid black circles, respectively. Evolved populations at different timepoints were grown with AHL to measure relative GFP levels. Relative fluorescence normalized by OD is plotted vs. generations. Error bars represent one standard deviation from the mean of nine independently evolved populations. (C) This circuit repeatedly has a deletion between homologous repeated terminators after 30 generations in the high input evolved populations.

**Figure 2:**
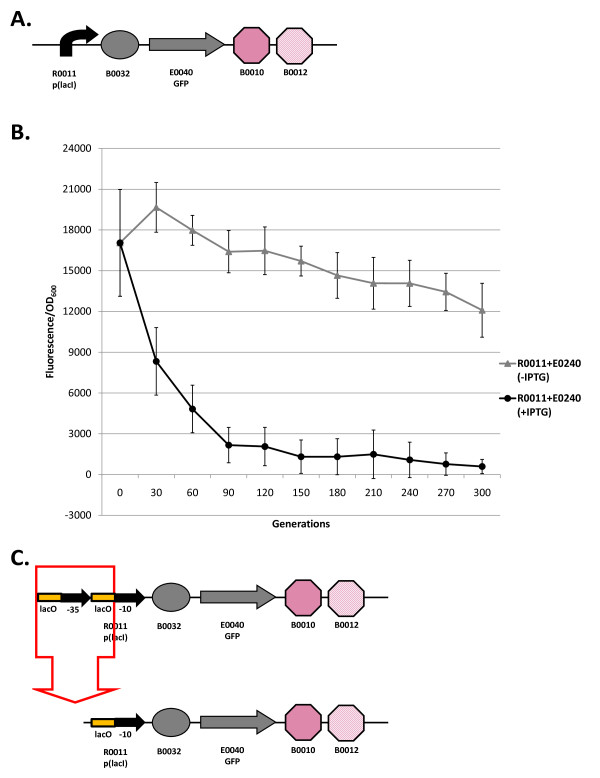
**Loss-of-function mutations and evolutionary stability dynamics in I7101**. (A) The I7101 genetic circuit. Symbols depict promoters (bent arrows), ribosome binding sites (ovals), coding sequences (arrows), and transcriptional terminators (octagons). I7101 consists of the promoter R0011 and the GFP-expressing element E0240 that is made up of the B0032 RBS, E0040 GFP coding sequence, and B0010-B0012 (B0015) transcriptional terminator. Since *lacI *is constitutively expressed from the chromosome, it represses GFP expression from the *lacI*-regulated promoter R0011. When the inducer Isopropyl-beta-D-thiogalactopyranoside (IPTG) is added to the media, it inhibits LacI and activates GFP expression. If no IPTG is in the media, the circuit is off. (B) Evolutionary stability dynamics of R0011 + E0240 evolved under low (-IPTG) and high (+IPTG) input conditions. Low and high input evolved populations are shown with solid gray triangles and solid black circles, respectively. Evolved populations at different timepoints were grown with IPTG to measure relative GFP levels. Relative fluorescence normalized by OD is plotted vs. generations. Error bars represent one standard deviation from the mean of nine independently evolved populations. (C) This circuit repeatedly has a deletion between homologous operators within R0011 after 90 generations in the high input evolved populations.

The two circuits we used to measure the evolutionary stability dynamics and determine the loss-of-function mutations were T9002 (Figure 1a) and I7101 (Figure 2a). T9002 is the Lux receiver circuit previously described [17] and expresses *luxR *that activates GFP expression when the inducer AHL is added to the media (see Figure 1 legend for details). I7101 has a *lacI*-regulated promoter and expresses GFP only when the inducer IPTG is added to the media since *lacI *is constitutively overexpressed from the chromosome in this particular strain (*Escherichia coli *MG1655 Z1). The evolutionary stability dynamics were measured by serial propagation with a dilution factor that allows for about 10 generations per day.

**Figure 3:**
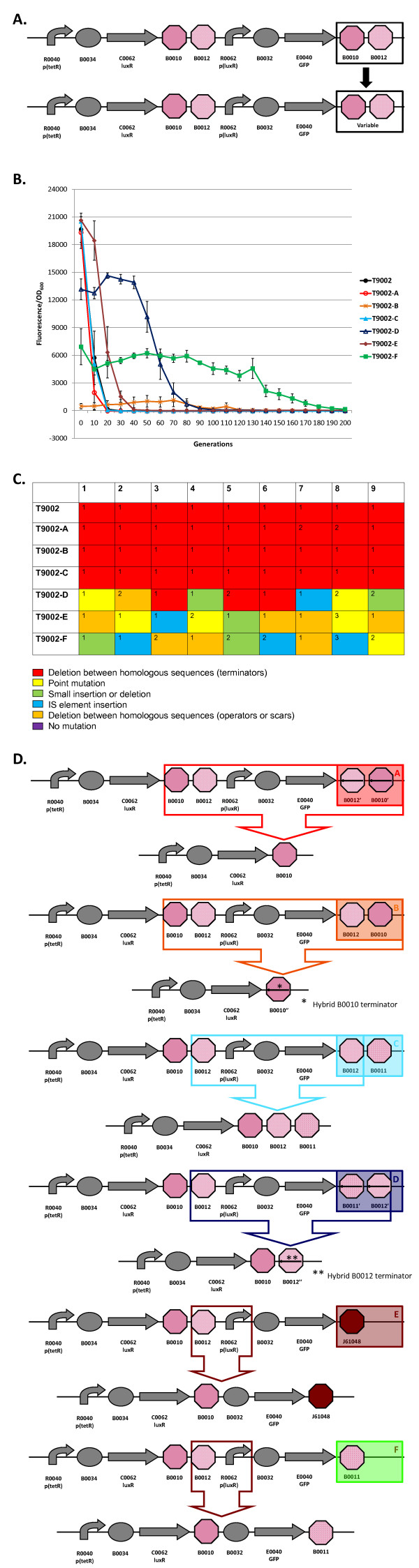
**Loss-of-function mutations and evolutionary stability dynamics in re-engineered T9002 circuits**. (A) T9002 re-engineering involves changing the second double transcriptional terminator with varying degrees of homology and orientation to the first double transcriptional terminator. (B) Evolutionary stability dynamics of T9002 (solid black circles) and T9002 re-engineered circuits (various shapes and colors) under high input (+AHL) conditions. Error bars represent one standard deviation from the mean of nine independently evolved populations. (C) Types of mutations in nine independently evolved populations. For nine independently evolved populations, colored boxes correspond to the mutation legend below the table. The most common mutation for a particular type of mutation is labeled with "1" in the boxes above and less common mutations are labeled with increasing numbers. (D) Most common loss-of-function mutations that inactivated the re-engineered T9002 circuits. See Additional File 1, Supplementary Table S1 for mutation details.

Figure 1b shows the evolutionary stability dynamics of the T9002 circuit propagated in high input (with AHL) and low input (without AHL) conditions. From different timepoints in the experiment, the low and high input populations were induced with AHL to measure their normalized expression (here measured by fluorescence divided by cell density) over time. The low input evolved populations slowly lose their function to about 50% of the maximum after 300 generations. The evolved populations in high input conditions rapidly lose their function in less than 30 generations (the dynamics of this evolutionary stability are described below in Figure 3). No functional clones were observed after 30 generations as determined by measurement of individual colonies. The mutation that repeatedly causes loss-of-function in the high input evolved populations is a deletion between two homologous transcriptional terminators (Figure 1c), the same mutation described in [17]. This mutation evidently occurs at such a high rate that mutants quickly take over the population. In fact, Canton *et al *(2008) [17] were unable to isolate a population derived from a single isolate that did not already carry the deletion. The mutant plasmid was transformed back into the progenitor and was shown not to fluoresce after induction with AHL. In this initial study we also tested the evolutionary stability of a BioBrick engineered version of the repressilator circuit [1]. We could not measure its function over time due to unstable GFP expression at the population level, but found that the circuit repeatedly had a deletion between homologous *tetR *promoters.

Figure 2b shows the evolutionary dynamics of the I7101 circuit propagated in high (with IPTG) and low input (without IPTG) conditions. The evolved populations in low input conditions lose about 70% of their function over 300 generations. The high input evolved populations lose about half their function in 30 generations and nearly all function after 300 generations. For this circuit, the loss-of-function is repeatedly a deletion between two homologous operator sequences in the promoter (Figure 2c). The mutant plasmid was transformed back into the progenitor and was shown not to fluoresce when induced with IPTG. This initial study suggests that the use of repeated parts in synthetic circuits should be avoided due to the high mutation rate. Also, there is a high metabolic load associated with the expression of genetic circuits on high-copy plasmids since keeping these circuits off substantially improves evolutionary stability.

### Evolution experiment with re-engineered circuits

Based on the results of the previous experiments, we re-engineered the T9002 and I7101 circuits to test various predictions of evolutionary stability and mutational predictability. For the T9002 circuit, the loss-of-function mutation was repeatedly a deletion between two homologous transcriptional terminators. Mutations and genetic rearrangements can occur due to misalignment of homologous sequences during replication (termed "replication slippage") [27]. Deletion mutations between repeated sequences are known to be dependent upon repeat length, proximity, and homology level [28]. These deletions are *recA*-independent if the repeat length is less than 200 bp [27,29], as is the case with the repeated terminators in the T9002 circuit. Thus, we re-engineered the last terminator of T9002 with various terminators available in the Registry to measure the effect of terminator homology level and orientation with evolutionary stability. We predicted that we could increase evolutionary robustness by decreasing the mutation rate of this deletion. Furthermore, although there have been several studies on recombination between repeated sequences, this phenomenon has not been studied in the context of synthetic biology using genetic circuits constructed from BioBricks. For instance, we do not know the effect of using various BioBrick terminators with different homology levels in the same circuit. The use of different terminators will become increasingly important when more complex circuits are constructed and BioBricks become even more widespread in the community. Also, many of the studies on recombination between repeated sequences use antibiotic resistance genes to measure recombination rates and may not relate to actual functioning circuits.

We also re-engineered the I7101 circuit in two separate ways. The first was to re-engineer I7101 with various promoters from a promoter library to test whether evolutionary stability is inversely correlated with promoter strength. The second was to insert a kanamycin resistance gene (*kanR*) within the circuit to put a selective pressure on the promoter. We engineered two versions of the KanR circuit, one as a GFP-KanR fusion protein with a glycine-serine linker and another where *kanR *is polycistronically expressed with GFP. Finally, we tested the effect of inducible vs. constitutive expression on evolutionary stability with two circuits having different *lacI*-regulated promoters.

Since we learned from previous experiments that evolutionary stability dynamics of genetic circuits have high variability between replicate populations, we evolved nine independent populations of each re-engineered circuit for at least 300 generations. Three experimental replicate populations of three independent tranformants were used to test for independent mutational events. A single transformant may have a mutation at a low level that will eventually sweep through the population, so if only one transformant was used, the same mutation may show up in all replication populations. For each of the nine populations in every circuit, the evolutionary half-life was measured to quantitate the number of generations until the expression level had decreased to half of its initial level (Table 1). Plasmids from a single clone from each evolved population were then sequenced after the population level had decreased to below 10% of the original expression level, or after 500 generations, whichever came first. Additional File 1, Supplementary Table S1 shows all mutations for each population in every circuit.

**Table 1:** Evolutionary half-life of various genetic circuits

Circuit	Evolutionary Half-life	SD
T9002	7.056	3.657

T9002-A	5.563	1.687

T9002-C	6.650	3.250

T9002-D	56.986	6.188

T9002-E	16.701	4.966

T9002-F	125.443	47.664

R0011 + E0240	19.833	3.818

R0011 + E0240 (inducible)	31.782	17.129

R0010 + E0240	42.363	14.729

R0010 + E0240 (inducible)	45.233	56.350

R0040 + E0240	59.838	13.445

J23101 + E0240	96.694	28.880

J23102 + E0240	36.428	13.338

J23105 + E0240	18.686	11.430

J23151 + E0240	62.844	27.168

R0010 + E0240 kanR polycistronic	71.000	29.031

R0010 + E0240 kanR polycistronic (+kan)	78.091	36.084

R0010 + E0240 kanR fusion	57.757	38.750

R0010 + E0240 kanR fusion (+kan)	66.252	54.056

The mean evolutionary half-life for nine independently evolved populations is shown for each genetic circuit with the standard deviation (SD). See the Methods section for details.

### Re-engineered T9002 circuits with different transcriptional terminators: loss-of-function mutations and evolutionary stability dynamics

Figure 3a shows the schematic for re-engineering the last transcriptional terminator in the T9002 circuit. The evolutionary stability dynamics for six re-engineered T9002 circuits and the original T9002 circuit are shown in Figure 3b. Figure 3c shows the type of mutations that occurred in each of the nine replicate evolved populations. Finally, Figure 3d shows the most common mutations for each re-engineered circuit. The six re-engineered circuits are labelled T9002-A through T9002-F in Figure 3b and color-coded to correspond to the same circuit mutations shown in Figure 3d. These circuits were all propagated with the inducer AHL to allow for GFP expression. In the following paragraphs, the loss-of-function mutations and evolutionary stability dynamics for the original T9002 circuit and each re-engineered circuit will be described in detail.

T9002: The original T9002 circuit decreases rapidly to about 30% of the original level after only 10 generations and all function is lost by 20 generations (Figure 3b). The same deletion between homologous terminators as was observed in previous experiments (Figure 1c) was found in all nine replicate populations (Figure 3c). The evolutionary half-life of this circuit was found to be about 7.1 generations on average (Table 1).

T9002-A: The final double terminator in T9002 was re-engineered in the reverse complementary orientation (called B0025 in the Registry) to make T9002-A. The stability of this circuit has approximately the same expression level and stability dynamics as T9002, but has an evolutionary half-life of about 5.6 generations (Figure 3b, Table 1). This decreased stability may be because the terminator in the reverse orientation is more likely to cause replication slippage. Since the expression level is similar to T9002 and therefore the metabolic load should be roughly equivalent, the difference in stability is primarily due to an increased mutation rate. Seven of nine replication populations have a deletion between the first B0010 terminator and the reverse complement of B0010 (Figure 3c and 3d). This effect likely occurs because B0010 has a long hairpin structure, so one half of B0010 can interact with the other half of the reverse complementary B0010 sequence during DNA replication since they have perfect homology. Two of the nine populations had a deletion that formed a triple terminator of B0010-B0012-B0010 (Additional File 1, Supplementary Table S1).

T9002-B: The T9002-B circuit was re-engineered to re-arrange the B0010 and B0012 terminators to have B0012 first and then B0010. The re-arrangement decreases the expression level to almost zero initially and this expression drifts up over time and then decreases to zero (Figure 3b). For this circuit, evolutionary half-life measurements are essentially meaningless due to the randomness of low expression. Notice that other re-engineered T9002 circuits also have decreased expression levels relative to T9002, presumably due to weaker terminator hairpin structures having increased mRNA degradation [30] or transcriptional readthrough that can decrease plasmid copy number [31]. Others have observed that removal of transcriptional terminators can decrease expression levels in general [32]. All nine populations have the same deletion between B0010 terminators (Figure 3c and 3d). Because the B0010 terminator is an inexact hairpin (there are some mismatches), one half of the first B0010 interacts with the other half of the second B0010 terminator, causing a hybrid B0010 terminator (Figure 3d, Additional File 1, Supplementary Table S1).

T9002-C: The T9002-C circuit was re-engineered to have B0012 and B0011 as the final double terminator instead of B0010 and B0012. This circuit has nearly identical stability dynamics as T9002, with an evolutionary half-life of about 6.7 generations on average (Figure 3b, Table 1). All nine populations have the same deletion between B0012 terminators that make a triple terminator of B0010-B0012-B0011 (Figure 3c and 3d). Since the expression level and stability dynamics are roughly equivalent to T9002, the mutation rate between repeated B0010-B0012 terminators (129 bp) is probably about the same as between repeated B0012 terminators (41 bp). Interestingly, no significant stability difference was observed between T9002-C (41 bp homology) and T9002 or T9002-A (both 129 bp homology), despite having similar expression levels. This result suggests that shortening the repeated regions of homologous terminators did not increase evolutionary robustness, contrary to what we expected.

T9002-D: The T9002-D circuit has the same final B0012-B0011 terminator, but is the reverse complement of this sequence. The inclusion of this terminator decreases the initial expression level to about 65% of T9002 (Figure 3b). The evolutionary half-life of this circuit is about 57 generations (Figure 3b, Table 1). Also, the slope of the stability plot is decreased relative to other circuits with higher expression (T9002, T9002-A, T9002-C, and T9002-E) and the stability lag (time for expression to decrease to zero along the x-axis) is increased (Figure 3b). In contrast to other circuits with repeated terminators, only 3/9 have deletions between homologous terminators forming a hybrid B0012 terminator (Figure 3c and 3d). This result is probably because, unlike T9002-C, the second B0012 is the reverse complement, and therefore the only homology in this circuit is the 8-bp of hairpin structure having complementary sequences; the rest of the terminator has a loop structure of non-complementary sequences. In other words, B0010 has sufficient homology in either the forward or reverse orientation to cause replication slippage, but in B0012 replication slippage is more likely to occur only in the forward orientation. The other mutations in this circuit are composed of point mutations, short insertions or deletions, deletions between scar sequences, or insertion sequence (IS) mutations (Figure 3c, Additional File 1, Supplementary Table S1).

T9002-E: The T9002-E circuit, like the T9002-F circuit, was re-engineered to have no homology between terminator sequences. This circuit has the highest initial expression level on average probably because J61048 is a very strong terminator, but has similar expression relative to the T9002, T9002-A, and T9002-C circuits (Figure 3b). Its evolutionary half-life is about 16.7 generations (Figure 3b, Table 1). Thus, relative to other circuits with the similar expression levels, it is the most evolutionary robust circuit, having over 2-fold higher stability than T9002. When the evolutionary half-life is measured for the nine replicate populations, this evolutionary half-life difference compared to T9002 is highly significant (one-tailed t-test, p = 0.0003). Notice that the stability slope is similar to T9002, T9002-A, and T9002-C circuits, but the stability lag is increased by about 10 generations. This difference in lag is likely due to a decreased mutation rate since mutations are more random compared to the other similar expression-level circuits (Figure 3c, Additional File 1, Supplementary Table S1). The most common mutation is a deletion between repeated scar sequences that removes the *luxR *promoter and effectively inactivates the circuit function (Figure 3d).

T9002-F: The T9002-F circuit was re-engineered with the B0011 terminator, so it also has no homology between terminator sequences. The B0011 is evidently a weak terminator since its initial expression level is about 4-fold lower than T9002. Its stability dynamics show that it is the most evolutionary robust of the re-engineered T9002 circuits, with an evolutionary half-life of about 125 generations (Figure 3b, Table 1). This result indicates that decreasing homology levels and expression through terminator re-engineering increased the evolutionary half-life of this circuit over 17-fold relative to T9002. Like T9002-E, the mutations in each of the nine populations are mostly random (Figure 3c, Additional File 1, Supplementary Table S1). Also like T9002-E, the most common mutation is a deletion between repeated scar sequences that removes the *luxR *promoter driving GFP expression (Figure 3d). Since T9002-E and T9002-F likely have similar mutation rates with zero terminator homology, the large stability difference between these circuits can be explained by expression levels alone.

Overall, excluding T9002-B, three of the five re-engineered T9002 circuits are more evolutionary robust than the original circuit. The order of evolutionary robust genetic circuits is: T9002-F > D > E > A = C = T9002. This increase in evolutionary robustness can be attributed to decreased expression levels (due to the terminator re-engineering) and to decreased mutation rate between homologous transcriptional terminators. The re-engineered circuits with homologous transcriptional terminators almost always have deletions between homologous regions, whereas circuits without homology have mutations in other locations in the circuit. Re-engineering this circuit to remove all homology effectively removes a certain class of mutations from occurring. The T9002-E circuit is more evolutionary robust than other circuits with similar expression levels likely due to decreased mutation rate alone. Thus, evolutionary robustness can be increased by removing long repeated sequences from genetic circuits, but even short 8-bp scar sequences have the potential for replication slippage.

### Re-engineered I7101 circuits with different promoters: loss-of-function mutations and evolutionary stability dynamics

**Figure 4:**
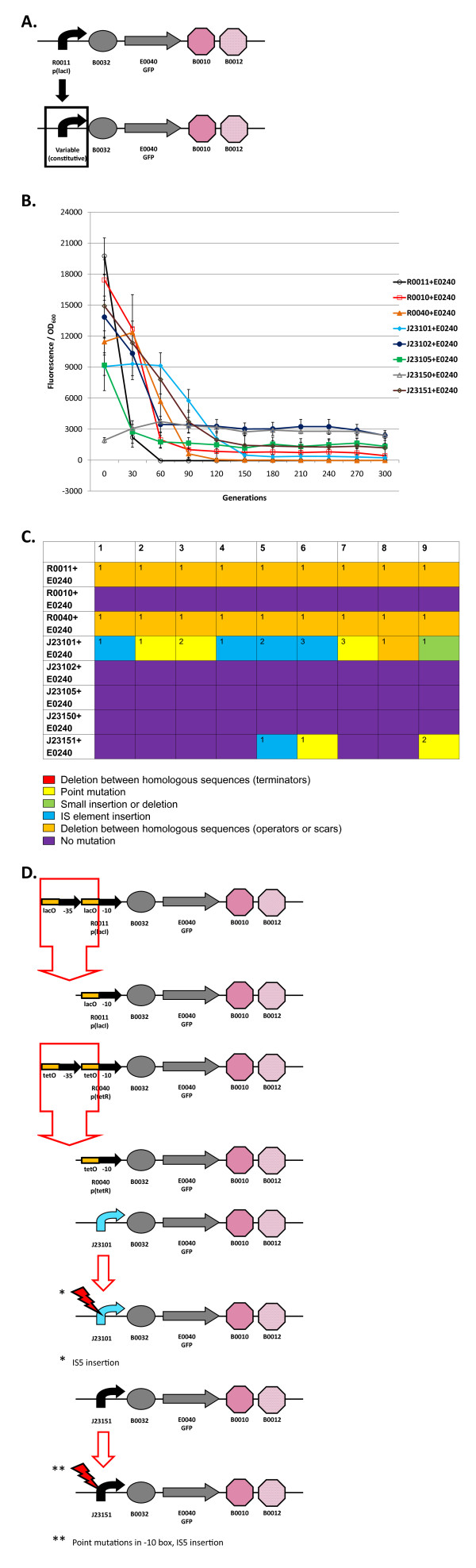
**Loss-of-function mutations and evolutionary stability dynamics in re-engineered I7101 circuits **(A) I7101 (R0011 + E0240) re-engineering involves swapping out the R0011 promoter. (B) Evolutionary stability dynamics of R0011 + E0240 (open black circles) and re-engineered circuits (various shapes and colors) under constitutive expression. Error bars represent one standard deviation from the mean of nine independently evolved populations. (C) Types of mutations in nine independently evolved populations. For nine independently evolved populations, colored boxes correspond to the mutation legend below the table. The most common mutation for a particular type of mutation is labeled with "1" in the boxes above and less common mutations are labeled with increasing numbers. (D) Most common loss-of-function mutations that inactivated the re-engineered I7101 circuits. See Additional File 1, Supplementary Table S1 for mutation details.

Figure 4 shows the re-engineering scheme for the I7101 circuit with different promoters (Figure 4a), evolutionary stability dynamics for these re-engineered circuits (Figure 4b), mutations found in the nine evolved populations (Figure 4c), and the most common mutation for each re-engineered circuit (Figure 4d). These circuits were constitutively expressed (no inducer was required to activate the circuit). Many of these promoters were used in the promoter measurement kit, an effort to standardize promoter measurements between promoters [33]. This previous study used medium-copy plasmids whereas we used high-copy plasmids and different environmental conditions. The loss-of-function mutations (if any) and evolutionary stability dynamics for the original I7101 circuit and each re-engineered circuit are described in detail below.

I7101 (R0011 + E0240): The evolutionary stability of the original I7101 circuit was measured constitutively (unlike Figure 2 with inducible expression) and is shown in Figure 4b. The evolutionary half-life of this circuit is about 20 generations (Table 1) and reaches zero after 40 generations (plotted every 30 generations for clarity). Not surprisingly, all nine populations have the same deletion between repeated operator sequences in the promoter (Figure 4c and 4d).

R0010 + E0240: When the R0011 promoter is swapped out for R0010, the wildtype *lacI*-regulated promoter without repeated operator sequences, the initial expression level decreases slightly (Figure 4b). The evolutionary half-life of this circuit is about 42 generations (Table 1) and roughly double that of R0011 + E0240. This circuit is more evolutionary robust than R0011 + E0240 probably due to a combination of decreased expression level and mutation rate. Also, this circuit never reaches zero expression. When this circuit was sequenced, surprisingly no mutations were found in any of the nine populations (Figure 4c). However, expression levels decreased due to unknown mutations in the chromosome (Additional File 1, Supplementary Material).

R0040 + E0240: R0040 is a *tetR*-regulated promoter with repeated *tetO *operator sequences. This circuit's evolutionary half-life is about 60 generations (Figure 4b, Table 1). Unsurprisingly, all nine populations have a deletion between the repeated operator sequences, similar to that of R0011 + E0240 (Figure 4c and 4d).

J23101/J23102/J23105/J23150/J23151 + E0240: All five of these circuits have very different initial expression levels and evolutionary half-lives, but are similar in that they maintain relatively high expression levels after an initial decrease except for J23150 + E0240 that maintains a relatively constant level (Figure 4b, Table 1). Like R0010 + E0240, three circuits do not have any mutations in all nine populations.

In summary for this section, re-engineered I7101 circuits have a large amount of variation in stability. This variation is probably mostly due to differences in expression level, but mutation rate also contributes to this variability. We expected that promoter strength to be inversely proportional to evolutionary half-life, and this hypothesis was tested in the Evolutionary half-life measurements of individual populations section (Additional File 1, Supplementary Material). Only circuits with repeated operator sequences reached zero expression, probably because the deletion removes the entire -35 region and therefore no leaky expression can occur. Promoters without repeated operator sequences should be used to maximize evolutionary stability.

### Re-engineered I7101 circuits with a kanamycin resistance gene: loss-of-function mutations and evolutionary stability dynamics

**Figure 5:**
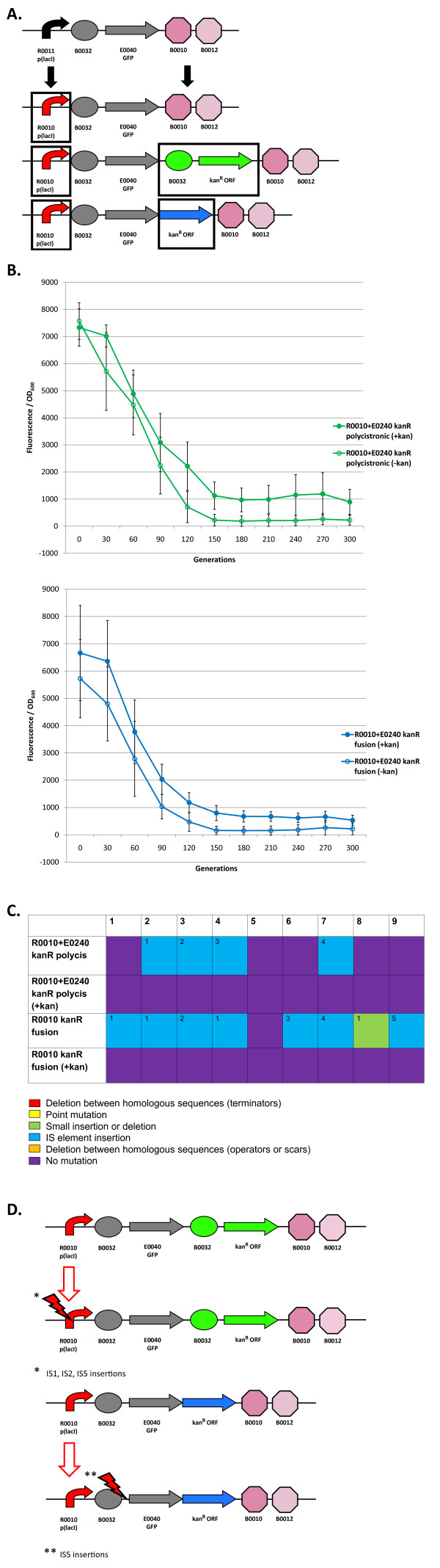
**Loss-of-function mutations and evolutionary stability dynamics in re-engineered I7101 circuits with a kanamycin resistance gene**. (A) I7101 re-engineering with the addition of a kanamycin resistance (*kanR*) gene. First the R0010 promoter was added instead of R0011 (top). Then, this circuit was re-engineered to polycistronically transcribe *gfp *and *kanR *separately into separate GFP and KanR proteins (middle) and to express a GFP-KanR fusion protein (bottom). (B) Top panel shows the evolutionary stability dynamics of R0010 + E0240 *kanR *polycistronic circuits propagated with kanamycin (solid green circles) and without kanamycin (open green circles). Bottom panel shows the evolutionary stability dynamics of R0010 + E0240 *kanR *fusion circuits propagated with kanamycin (solid blue circles) and without kanamycin (open blue circles). R0010 + E0240 and R0011 + E0240 evolutionary stability dynamics are shown in Figure 4. Error bars represent one standard deviation from the mean of nine independently evolved populations. (C) Types of mutations in nine independently evolved populations. For nine independently evolved populations, colored boxes correspond to the mutation legend below the table. The most common mutation for a particular type of mutation is labeled with "1" in the boxes above and less common mutations are labeled with increasing numbers. (D) Most common loss-of-function mutations that inactivated the re-engineered I7101 circuits with a kanamycin resistance gene. See Additional File 1, Supplementary Table S1 for mutation details.

We also re-engineered the I7101 circuit to have a kanamycin resistance gene (*kanR*) in order to put a selective pressure on the promoter (Figure 5a). Both a GFP-KanR fusion coding sequence and polycistronic transcribed coding sequence were engineered (Figure 5a). We also swapped out the R0011 promoter with the R0010 promoter since it was found to be more evolutionary robust than R0011 (Figure 5a). The other parts of this figure show the evolutionary stability dynamics for these re-engineered circuits (Figure 5b), mutations found in the nine evolved populations (Figure 5c), and most common mutation for each re-engineered circuit (Figure 5d). These circuits were constitutively expressed and the KanR circuits were propagated with and without kanamycin (kan) in the media. We predicted that the kan propagated circuits would be more evolutionary robust than those without kan in the media.

The upper panel of Figure 5b shows the evolutionary stability dynamics for the R0010 + E0240 *kanR *polycistronic circuits with and without kan in the media. Introduction of the *kanR *coding sequence into the R0010 + E0240 circuit lowers expression by about 60% (Figure 4b and Figure 5b, upper panel). This lowered expression may be due to competition between the RBS used for GFP translation vs. the RBS used for KanR translation. This difference in expression complicates any useful comparisons between the R0010 + E0240 circuit and the R0010 + E0240 *kanR *polycistronic circuit. However, since the KanR circuit was measured with and without kan in the media, we can determine the effect the kanamycin is having on the circuit independent of expression levels. The KanR circuit propagated with kan has an evolutionary half-life of about 78 generations whereas without kan the half-life is 71 generations, but this result is not statistically significant (one-tailed t-test, p = 0.0896). However, the circuit propagated with kan remains at a higher level over time (Figure 5b, upper panel).

The lower panel of Figure 5b shows the same comparison between circuits except with the GFP-KanR fusion version of the circuit. Introduction of the *kanR *fusion coding sequence also lowers expression by about 60% and this may be due to folding issues in the GFP-KanR fusion protein. Similar to the polycistronic version of the KanR circuit, the fusion version also only has an evolutionary half-life difference of less than 10 generations when propagated with kan compared to without kan in the media (Table 1 and Figure 5b, lower panel). Again, this result is not statistically significant (one-tailed t-test, p = 0.1174). Like the polycistronic version of this circuit, propagation in kan causes evolutionary stability to remain at a higher level over time (Figure 5b, lower panel).

Figure 5c shows the mutations found in each of the nine populations. Interestingly, 4/9 of the KanR polycistronic circuits and 7/9 of the KanR fusion circuits propagated without kan in the media have IS mutations at the generation 200 timepoint. The KanR circuits propagated with kan in the media and the R0010 + E0240 circuit have no mutations at this timepoint. However, after propagating these KanR circuits for 500 generations, 2/9 populations had mutations in the promoter that were not IS mutations. Evidently, mutations on the chromosome confer resistance to kanamycin because introduction of these mutated circuits back into the progenitor does not make these cells kan resistant.

Since only KanR circuits propagated without kan in the media have IS mutations, propagating strains carrying the metabolic load of express resistance to both ampicillin (encoded on the plasmid backbone) and kanamycin could have caused a significant amount of cellular stress and triggered IS transposition. Some evidence suggests that IS transposition can occur in response to stress [34-38], but it also occurs in non-stressful conditions as well [26,38,39]. IS mutations probably occurred in the KanR circuits propagated with kan in the media as well, but cells carrying these circuits were selected against. Additional experiments partially explain why KanR circuits propagated with kan lose expression over time without having mutations in the circuit itself (Additional File 1, Supplementary Material).

The IS mutations in the KanR circuits propagated without kan in the media consisted of IS1, IS2, and IS5 mutations (Figure 5d, Additional File 1, Supplementary Table S1). A hotspot for IS5 mutations "C(T/A)A(G/A)" [40,41] often occurs in the "CTAG" portion of the scar sequence between either the promoter and RBS ("TA**CTAG**AG") or RBS and coding sequence ("TA**CTAG**"). This one unexpected result may be unfortunate for genetic circuits assembled with BioBricks if the host strain carries IS5 elements and if evolutionary stability is an issue. Also, since the IS5 mutations in these circuits were located in a position just downstream of the promoter, we thought that the transposase gene within the IS element might be transcribed and translated. However, the orientation of these IS insertions in these circuits would not allow for expression to occur. Unlike IS5 which has a defined hotspot, the IS1 and IS2 mutations are more random, but appear to transpose in A-T rich regions (Additional File 1, Supplementary Table S1).

**Figure 6:**
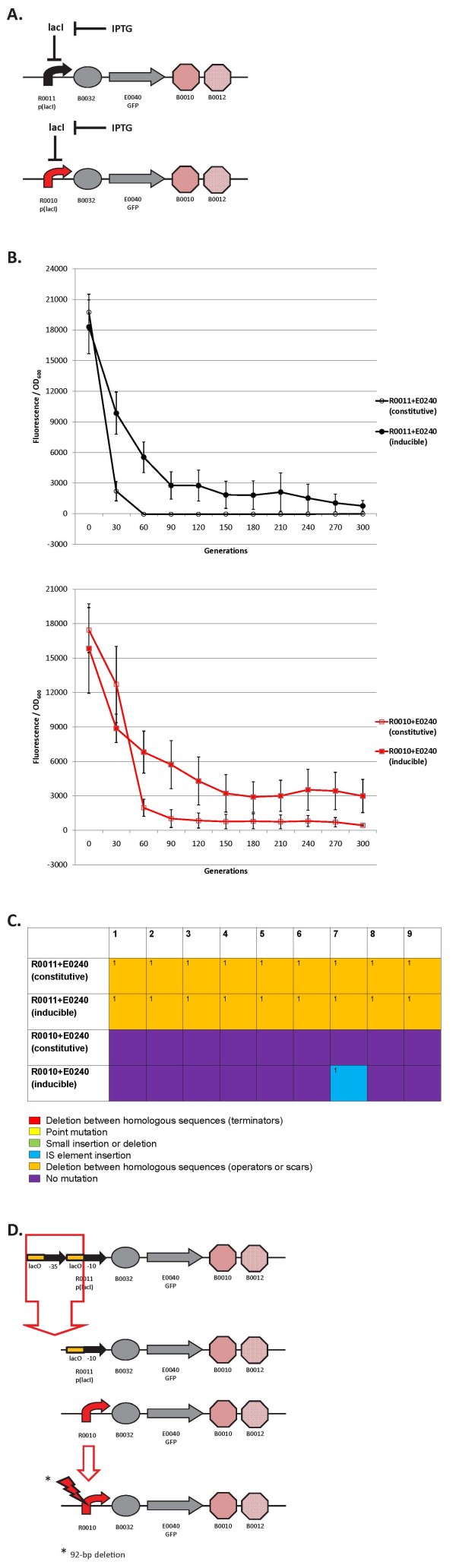
**Loss-of-function mutations and evolutionary stability dynamics in two *lacI*-regulated circuits under constitutive vs. inducible expression**. (A) Regulation of inducible R0011 + E0240 and R0010 + E0240 circuits. LacI represses transcription of GFP and IPTG de-represses the circuit to allow for GFP expression. (B) Top panel shows the evolutionary stability dynamics of constitutive R0011 + E0240 (open black circles) and inducible R0011 + E0240 (solid black circles). Bottom panel shows the evolutionary stability dynamics of constitutive R0010 + E0240 (open red circles) and inducible R0010 + E0240 (solid red circles). Error bars represent one standard deviation from the mean of nine independently evolved populations. (C) Types of mutations in nine independently evolved populations. For nine independently evolved populations, colored boxes correspond to the mutation legend below the table. The most common mutation for a particular type of mutation is labeled with "1" in the boxes above and less common mutations are labeled with increasing numbers. (D) Most common loss-of-function mutations that inactivated the R0011 + E0240 and R0010 + E0240 inducible and constitutive circuits. See Additional File 1, Supplementary Table S1 for mutation details.

Overall, the use of a kanamycin resistance gene within the circuit does not significantly increase evolutionary stability. Although propagation with kanamycin in the media may put a selective pressure on the promoter, other mutational targets evidently can decrease expression of the circuit over time (Additional File 1, Supplementary Material). IS mutations are responsible for loss-of-function in the KanR circuits propagated without kan. Since there are relatively few IS mutations in circuits without *kanR *(Figures 3c, 4c, 5c, and 6c), this circuit may induce IS transposition bursts.

As a final note for this section, we also tried to insert *kanR *into the T9002 circuit that would be polycistronically transcribed with GFP. The cells carrying this circuit did not grow well when AHL was added to the media. Expressing *kanR *using the strong *luxR *promoter may have been somewhat toxic to the cells. Decreasing the strength of the RBS that controlled the translation of *kanR *may have helped to avoid this growth deficiency.

### Constitutive vs. inducible expression between two *lacI*-regulated circuits: loss-of-function mutations and evolutionary stability dynamics

We also propagated two *lacI*-regulated circuits (R0011 + E0240 and R0010 + E0240) with inducible vs. constitutive expression. We speculated that there might be a difference in stability since the constitutively expressed circuits may have a larger metabolic load. In the inducible circuits, the LacI protein may rebind to the DNA after some time and repress transcription. For inducible expression, we propagated the circuits in MG1655 Z1 strains that constitutively overexpresses *lacI *from its chromosome. Constitutively expressed circuits were propagated in the normal MG1655 strain that does not overproduce LacI. Figure 6 shows the regulation of the inducible circuits (Figure 6a), evolutionary stability dynamics for these re-engineered circuits (Figure 6b), mutations found in the nine evolved populations (Figure 6c), and most common mutation for each re-engineered circuit (Figure 6d).

For both circuits, evolutionary stability is increased on average when inducible expression is used compared to constitutive expression (Figure 6b). The evolutionary half-life of the R0011 + E0240 circuit under inducible control is about 32 generations vs. 20 for constitutive expression (one-tailed t-test, p = 0.0422). For R0010 + E0240, the inducible evolutionary half-life is 45 generations vs. 42 generations for constitutive expression (one-tailed t-test, p = 0.1083). Therefore, the evolutionary half-life is only significantly increased for one of the two circuits, but on average inducible expression increases evolutionary robustness. For the R0011 + E0240 circuit under inducible expression, by 30 generations the circuit is already about 4-fold higher even though it had a lower initial expression (Figure 6b, upper panel). After 60 generations, the circuit under constitutive expression has lost all function and the inducible circuit has 25% of its original expression level (Figure 6b, upper panel). Furthermore, the slopes are quite different between inducible and constitutive expression. The inducible R0010 + E0240 circuit is over 3-fold higher after 60 generations compared to the circuit under constitutive expression (Figure 6b, lower panel). The reason that inducible circuits may be more evolutionary robust than constitutive circuits is that there may a greater metabolic load to constantly express GFP. Perhaps in the inducible circuits, LacI eventually re-inhibits GFP expression and decreases this metabolic load.

The mutations in both circuits are similar for both inducible and constitutive expression (Figure 6c). For R0011 + E0240, all have the same mutation between repeated operator sequences (Figure 6c and 6d). For R0010 + E0240, only one mutation was observed in one inducible population, an IS element insertion (Figure 6c and 6d), indicating that differences in inducible vs. constitutive expression largely do not change the type of mutations that occur.

### Evolutionary half-life vs. initial expression level in T9002, T9002-E, R0011 + E0240, and R0010 + E0240 circuits evolved with different inducer concentrations

From the results in previous sections (Figures 3, 4, 5, 6, Table 1), we noticed that circuits with a high initial expression level tended to have low evolutionary stability. Also, particular circuits with high mutation rates had lower stability compared to circuits with lower mutation rates. To test the relationship between initial expression level and evolutionary half-life directly, we evolved four circuits for up to 300 generations propagated with different inducer concentrations. We tested initial expression level vs. evolutionary half-life in T9002 (high mutation rate) vs. T9002-E (lower mutation rate) using different AHL concentrations. We also tested initial expression level vs. evolutionary half-life in R0011 + E0240 (high mutation rate) vs. R0010 + E0240 (lower mutation rate) using different IPTG concentrations in the Z1 strain.

**Figure 7:**
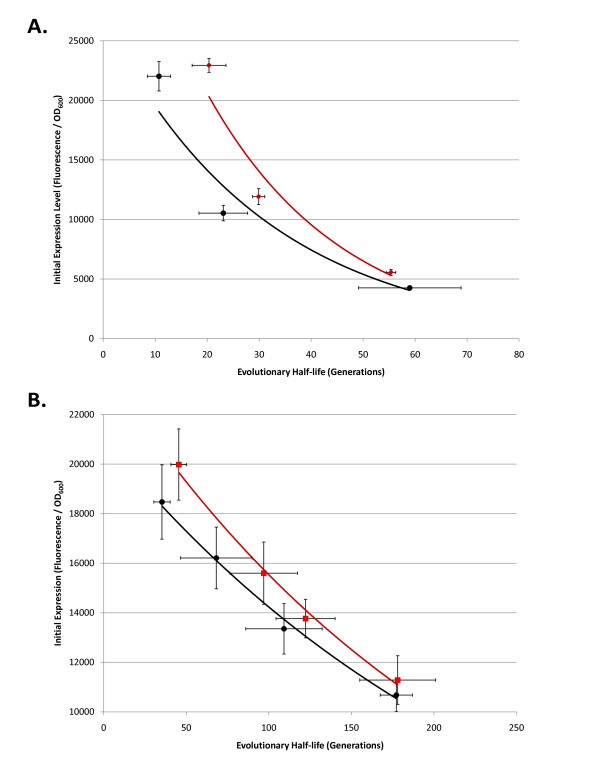
**Evolutionary half-life vs. initial expression level in T9002, T9002-E, R0011 + E0240, and R0010 + E0240 circuits evolved with different inducer concentrations**. (A) Evolutionary half-life vs. initial expression level is plotted in T9002 (solid black circles) and T9002-E (solid dark red diamonds) circuits evolved with different AHL concentrations. An exponential fit for the mean of each evolutionary half-life vs. initial expression data point is shown by the black line. Error bars for both the x and y-axis represent one standard deviation from the mean of eight independently evolved populations. (B) Evolutionary half-life vs. initial expression level is plotted in R0011 + E0240 (solid black circles) and R0010 + E0240 (solid red circles) circuits evolved with different IPTG concentrations. An exponential fit for the mean of each evolutionary half-life vs. initial expression data point is shown by the black line. Error bars for both the x and y-axis represent one standard deviation from the mean of eight independently evolved populations.

The results of these experiments are shown in Figure 7. Figure 7a shows the mean initial expression level vs. mean evolutionary half-life for eight replicate populations from three different AHL concentrations in T9002 (black) and T9002-E (red). An exponential fit of these mean data points gives a much higher r^2 ^value than a linear fit (> 0.1) in both cases. T9002 has an r^2 ^value of 0.954 compared to the r^2 ^value of 0.955 in T9002-E. The curve for T9002 is shifted to the left from T9002-E due to its higher mutation rate (expression alone cannot account for the shift), but as expression is decreased the evolutionary half-life difference between these two circuits also decreases. This decrease may be because at high expression levels, the fitness difference between the progenitor and mutant cells is the highest, and therefore mutants outcompete functional cells in the population more quickly.

Figure 7b shows the mean initial expression level vs. mean evolutionary half-life for eight replicate populations from four different IPTG concentrations in R0011 + E0240 (black) and R0010 + E0240 (red). The R0011 + E0240 exponential curve has an r^2 ^value of 0.993 whereas the r^2 ^value of R0010 + E0240 is 0.992. These r^2 ^values are also both higher than a linear fit (> 0.02). Similar to the T9002 vs. T9002-E curves (Figure 7a), the curve for R0011 + E0240 is also shifted to the left due to its higher mutation rate and the evolutionary half-life difference decreases at lower expression levels. This difference is not as apparent as in Figure 7a, but the curves may have been more similar if there were more data points for the IPTG-induced populations at lower expression levels (evolutionary stability was relatively stable at 300 generations for these populations). The other striking difference between Figures 7a and 7b is that the T9002 and T9002-E circuits have a much lower evolutionary stability than the R0011 + E0240 and R0010 + E0240 circuits for any particular expression level. This result could be for a number of reasons that include mutation rate and fitness differences between functional and non-functional cells (due to circuit length, activator vs. repressor regulation, or something particular to the strong *luxR *promoter).

We also performed a regression on the individual T9002, T9002-E, R0011 + E0240, and R0010 + E0240 initial expression level vs. evolutionary half-life for independently evolved populations (Additional File 1, Supplementary Figures S2 and S3). While the r^2 ^values are not as high for individual data points compared to means (Figure 7) and range from about 0.6 to almost 0.9, the regressions are highly significant (p < 0.0001). For these regressions, an exponential fit is always higher than a linear fit in all cases, suggesting that on average, evolutionary half-life exponentially decreases with increasing expression levels. We also performed a regression on the initial expression level vs. evolutionary half-life for independent populations (shown in Figures 3, 4, 5, 6, Additional File 1, Supplementary Figures S4, S5, S6, S7, S8).

## Discussion

Genetic circuits lose function over evolutionary time and are found to have a wide variety of mutations that cause their loss-of-function. Circuits with repeated sequences almost always have deletions between these sequences. These repeated sequences include transcriptional terminators, entire promoters, operator sequences within promoters, and occasionally between 8-bp scar sequences. In one re-engineered T9002 circuit, shortening the length of homology from 129 bp to 41 bp did not significantly increase evolutionary stability. Stability was only increased when there was no homology whatsoever between transcriptional terminators. Mutations between repeated sequences without perfect homology in the case of some re-engineered T9002 circuits are usually, but not always predictable.

In circuits without repeated sequences, mutations are more random between evolved replicate populations. Mutations that remove promoter function are most often selected for among all the genetic circuits tested. This result is likely because promoter mutations remove the metabolic load at both the transcriptional and translational levels. Mutations within RBSs are not found and mutations in coding sequences are rare except when that coding sequence is an activator of transcription downstream (as in the case of the *luxR *coding sequence in T9002). In the case of T9002, removing homology between transcriptional terminators only shifts the mutation to one that often removes function of the *luxR *promoter or *luxR *coding sequence instead. A similar story is seen with the KanR circuits. Even when a kanamycin resistance gene is inserted within the circuit and cells with this circuit are propagated with kanamycin to select for promoter function, mutations in the chromosome are selected for instead. Thus, without a selective pressure, removing the possibility of a mutation in one location only causes a mutation in another location. However, if this prevention lowers the mutation rate for a particular mutation, then evolutionary stability can be increased significantly, as shown for the T9002-E circuit.

What is needed is a method to predict the evolutionary stability of circuits from the properties of their parts, but the emergent behaviors of circuits will likely make prediction difficult. At the very least, publishing the evolutionary stability properties of simple circuits in future data sheets may allow engineers to calculate the expected evolutionary stability of more complex circuits. This calculation would likely require software (such as [42]) and mathematical modelling [43] that analyzes each part individually and the entire DNA sequence as a whole to determine the expected evolutionary stability. This calculation would also require standardization for methods to measure evolutionary stability and methods described here are not necessarily the best way. On the other hand, more general methods may be developed that focus less on design of the circuit and more on design of the environment to impose a selective pressure for function of the circuit [44]. Design of a selective environment is ideal, but is difficult to do when the output of most circuits (e.g., GFP) is not linked to survival or growth rate. A cell-sorter device that sorts between functional and non-functional cells may help with this issue, but may not be practical for performing routine experiments.

From our results of what types of mutations are selected for in genetic circuits and the evolutionary stability dynamics, a few simple design principles can be proposed when engineering circuits. The first principle is that high expression of genetic circuits comes with the cost of low evolutionary stability. Although exceptions to this rule certainly occur, a genetic circuit with high expression correlates with a large metabolic load and therefore is predicted to have decreased cellular fitness. When the fitness difference between the functional and non-functional cells in the population is large, evolutionary stability will decrease quickly. Therefore, the initial expression level of the circuit is likely to be a good predictor of evolutionary stability when a circuit with high mutational robustness is desired. Using a low or medium-copy plasmid will help with stability as long as the expression level does not need to be high. For more complex circuits where a high expression level is needed for proper functioning of the circuit, decreasing expression level then comes at the cost of changing the function of the circuit.

The second design principle is to avoid repeated sequences. This principle may be obvious, but nearly every circuit in the Registry with more than one coding sequence has repeated B0015 terminators. When a circuit has a high metabolic load (higher than T9002) and repeated sequences on a high-copy number plasmid, the circuit will almost always lose function during overnight growth (unpublished results). Re-engineering the T9002 circuit to have two different transcriptional terminators (T9002-E) does significantly increase evolutionary half-life over 2-fold and is independent of expression levels. However, since this circuit has high expression, this improvement only results in an increase of about 10 generations. Decreasing the expression level along with the mutation rate will increase the evolutionary half-life about 17-fold, as seen in the T9002-F circuit. This result suggests simple ways to increase evolutionary stability can be used without changing function of the circuit. For more complex circuits, the community will need to identify many more terminators than those that currently exist in the Registry to design circuits without repeated sequences.

The third design principle is that use of inducible promoters generally increases evolutionary stability. This principle may or may not be significant depending on the circuit used. Inducible circuits are likely more stable due to decreased metabolic load and are preferred since expression can be controlled and fine-tuned, though in some circumstances a constitutive promoter may be desired. Therefore, the use of inducible promoters can be thought of one extra precaution to maximize evolutionary stability, but expression levels and repeated sequences should first be considered.

We emphasize that the design principles proposed may not be general since only relatively simple circuits were tested in this study. Evolutionary stability should be measured in larger and more complex circuits to understand if these design principles apply. Furthermore, these simple design principles should not necessarily be all used simultaneously. A researcher may not want only to design circuits that have low expression, have no repeated regions, and use a promoter that is inducible. For instance, if recombination sites are needed in the circuit, then repeated or inverted sequences may be impossible to avoid. Besides the design for the proper function of the circuit, design for evolutionary robustness should be carefully considered. For this, we need to think about the probability of mutations occurring when the expression level, and therefore metabolic load, is high. In this study, removing repeated regions often shifts mutations to the promoter, and putting a selection on the promoter often shifts the mutation to the chromosome.

Thus, mutations are unavoidable without a selective pressure, but evolutionary stability can likely be improved in the future by better design of selective environments where the circuit is linked to survival and/or growth rate, understanding of mutation rates in genetic circuits, fitness differences between functional and non-functional cells, and improvements to the host strain that decrease mutation rates or buffer metabolic loads more efficiently. Another way to improve evolutionary stability is to engineer an error detection and correction circuit that will correct mutations, but will need careful design since this circuit itself will be prone to mutation. Designing evolutionary robust genetic circuits therefore is somewhat of an artform at the moment besides a few simple design rules, but should be seen as something the engineer can eventually control.

## Conclusions

A wide variety of loss-of-function mutations are observed in genetic circuits including point mutations, small insertions and deletions, large deletions, and insertion sequence (IS) element insertions that often occur in the scar sequence between parts. Promoter mutations are selected for more than any other biological part. Genetic circuits can be re-engineered to be more evolutionary robust with a few simple design principles: high expression of genetic circuits comes with the cost of low evolutionary stability, avoid repeated sequences, and the use of inducible promoters increases stability. Inclusion of an antibiotic resistance gene within the circuit does not ensure evolutionary stability.

## Methods

### Circuit engineering and use of strains

All circuits were either obtained from the Registry of Standard Biological Parts or engineered using the Clontech In-Fusion PCR Cloning Kit with the specific methods described in [19]. All circuits are encoded on the pSB1A2 plasmid, a high copy number plasmid (100-300 plasmids/cell) with an ampicillin resistance gene. Plasmids were transformed into strains via chemical transformation. *Escherichia coli *MG1655 was the strain used for constitutive expression and *Escherichia coli *MG1655 Z1 was used for inducible expression from *lacI*-regulated promoters since this strain is lacI^q ^(overexpresses *lacI *from its chromosome).

### Evolution experiment

For each engineered circuit, plasmid DNA that had been fully sequenced was transformed into either MG1655 or MG1655 Z1 competent cells. Three individual transformant colonies were grown overnight at 37°C shaking at 250 RPM in + 100 μg/mL ampicillin and supplemented with 50 μg/mL kanamycin for KanR circuits. Freezer stocks were saved of these cultures in 15% glycerol and stored at -80°C. These freezer stocks were streaked out on LB + 100 μg/mL ampicillin plates with appropriate inducer (1 × 10^-7 ^M AHL for T9002 circuits and 1 × 10^-3 ^M IPTG for LacI-regulated promoters) or antibiotic (50 μg/mL kanamycin for KanR circuits) and grown overnight at 37°C. Three colonies were chosen from each transformant (nine total colonies) and inoculated into 1.5 mL LB + 100 μg/mL ampicillin media in Eppendorf deep-well plates sealed with a Thermo Scientific gas permeable membrane to allow for maximum oxygen diffusion. T9002 circuit cultures were supplemented with the inducer 1 × 10^-7 ^M 3OC_6_HSL (AHL). KanR circuit cultures were supplemented with 50 μg/mL kanamycin. Also, *lacI*-regulated circuits under inducible expression were supplemented with 1 × 10^-3 ^M IPTG. T9002 and R0011 + E0240 were also evolved without inducer as controls. The R0011 + E0240 circuit with a mutation in the promoter was evolved to measure fluorescence background. Cultures were propagated with a serial dilution scheme using a 1:1000 dilution to achieve about 10 generations per day (log_2 _1000 = 9.97). Evolved populations were grown for 24 hours at 37°C shaking at 250 RPM. Freezer stocks (with 15% glycerol) of each population were saved at -80°C every day for future study. All replicate populations were evolved in parallel to minimize experimental variability.

### Evolutionary stability measurements

Every 24 hours, cell density (OD_600_) and fluorescence (excitation wavelength: 485/15, emission wavelength: 516/20) of evolved populations were measured in a Biotek Synergy HT plate reader. 24 hours was used as the measurement timepoint because the rate of change of GFP was closest to zero (i.e. closest to steady-state). Evolved populations thus spent about 8-12 hours in lag or exponential phase and the remaining time in stationary phase. For each timepoint, all populations were thoroughly mixed and 200 μl was transferred into black, clear-bottom 96-well plates (Costar). OD was subtracted from blank media to measure the OD without background. Fluorescence was subtracted from the R0011 + E0240 circuit with a mutation in the promoter to measure background fluorescence. Fluorescence was then divided by OD to measure the normalized expression (Fluorescence/OD_600_).

### Plasmid sequencing

At appropriate evolutionary timepoints, usually when circuit function had decreased to less than 10% of the original expression level, or 500 generations, the evolved populations were streaked out on LB + 100 μg/mL ampicillin plates, supplemented with 1 × 10^-7 ^M AHL (for T9002 circuits), 50 μg/mL kanamycin (for KanR circuits), or 1 × 10^-3 ^M IPTG (for *lacI*-regulated inducible circuits). Colonies were visualized for fluorescence on a Clare Chemical Dark Reader Transilluminator. Non-fluorescing colonies, or weakly fluorescing colonies if no non-fluorescing colonies were present, were grown overnight in 5 mL of LB + 100 ug/mL ampicillin. Plasmids were extracted using the Qiagen Miniprep Kit or glycerol stocks were sent to the University of Washington High-Throughput Genomics Unit facility http://www.htseq.org. Purified plasmid DNA was sequenced using the VF2 (5'-TGCCACCTGACGTCTAAGAA-3') and VR (5'-ATTACCGCCTTTGAGTGAGC-3') primers specific to the pSB1A2 vector (about 100 bp on either side of the circuit) or primers specific to the circuit.

### Quantitative analysis of evolutionary half-life

Evolutionary half-life was calculated for each independently evolved population. First, the slope and y-intercept were calculated using the two normalized expression (Fluorescence/OD_600_) data points on either side of the half maximum expression value on the evolutionary stability plot. A linear regression on those two data points was performed using the equation y = ax + b, where y = the half maximum initial expression, a = the slope of the two data points, b = the y-intercept of the two data points, and solving for x gives the evolutionary half-life. This method gave a very accurate half-life estimate in terms of generations and was a better estimate than using third-order polynomial equations which we also calculated.

### Experiment to measure evolutionary half-life vs. initial expression level in T9002, T9002-E, R0011 + E0240, and R0010 + E0240 circuits evolved with different inducer concentrations

This experiment was performed as described in the "Evolution experiment" section above except that eight replicate populations were propagated with different inducer concentrations. The results of this experiment are shown in Figure 7. For the T9002 and T9002-E circuits, the AHL concentrations were 1 × 10^-7 ^M (high expression level datapoint on the far left side of Figure 7a), 2 × 10^-9 ^M (medium expression level), and 1 × 10^-9 ^M (low expression level). For the R0011 + E0240 and R0010 + E0240 circuits, the IPTG concentrations were 5 × 10^-5 ^M (high expression datapoint on the far left side of Figure 7b), 3 × 10^-5 ^(medium-high expression level), 2 × 10^-5 ^(medium expression level), and 1 × 10^-5 ^(low expression level). The evolutionary half-life for individual evolved populations was determined as described in the "Quantitative analysis of evolutionary half-life" section above. For each inducer concentration, the mean evolutionary half-life vs. initial expression level data point was plotted. These data points were fit to an exponential curve since this relationship always had the highest r^2 ^value.

### Plasmid curing and re-transformation

To indirectly determine whether there are mutations on the chromosome, we first cured plasmids from evolved clones. Each evolved population was streaked out on to an LB plate and grown overnight at 37°C. Individual colonies were streaked on to both LB and LB + 100 μg/mL ampicillin plates where each colony was marked with a number. These plates were then grown overnight at 37°C. Colonies that were sensitive to ampicillin were grown overnight in LB and LB + 100 μg/mL ampicillin as a control to ensure sensitivity. These ampicillin sensitive cultures were made electrocompetent and saved in 15% glycerol stocks at -80°C. Plasmids were then re-transformed into these strains via electroporation and plated on selective media.

## Competing interests

The authors declare that they have no competing interests.

## Authors' contributions

SS conceived of the study, designed the experiments, engineered all the genetic circuits except for those described below, performed all the experiments, analyzed the DNA sequences, and analyzed all other data. BB engineered four of the six re-engineered T9002 circuits, JL engineered six of the eight I7101 circuits from a promoter library, and HS participated in the overall design of the study. All authors read and approved the final manuscript.

## Supplementary Material

Additional file 1 **Supplementary Material**. This file contains the genetic circuit mutations in all evolved populations, regressions of initial expression vs. evolutionary half-life measurements and additional experiments that test for mutations on the chromosome of evolved strains. Click here for file
