# Supplementary material for: Designing and engineering evolutionary robust genetic circuits
Source: J Biol Eng. 2010 Nov 1;4:12. doi: 10.1186/1754-1611-4-12 (PMC2991278; doi:10.1186/1754-1611-4-12)
Supplement: Additional file 1 — Supplementary Material. This file contains the genetic circuit mutations in all evolved populations, regressions of initial expression vs. evolutionary half-life measurements and additional experiments that test for mutations on the chromosome of evolved strains. [file 1754-1611-4-12-S1.PDF]

Supplementary Material

Circuit	Mutation	Generation	Description
T9002 (C1-C9)	Deletion	50	Remaining sequence: R0040-B0032-E0040-B0010-B0012
T9002-A (C1-C6, C9)	Deletion	50	Remaining sequence: R0040-B0032-E0040-B0010
T9002-A (C7, C8)	Deletion	50	Remaining sequence: R0040-B0032-E0040-B0010-B0012-B0010
T9002-B (C1-C9)	Deletion	150	Remaining sequence: R0040-B0032-E0040-B0010*
T9002-C (C1-C9)	Deletion	50	Remaining sequence: R0040-B0032-E0040-B0010-B0012-B0011
T9002-D (C1)	Point mutation	100	281 bp downstream of *luxR* start codon (G-->T)
T9002-D (C2)	Deletion	100	194-bp deletion between T9002 position 933-1126**
T9002-D (C3, C6)	Deletion	100	Remaining sequence: R0040-B0032-E0040-B0010-B0012***
T9002-D (C4)	Small deletion	100	1-bp deletion 527 bp downstream of *luxR* start codon
T9002-D (C5)	Deletion	100	Remaining sequence: R0040-B0032-E0040-B0010-B0012****
T9002-D (C7)	IS5	100	257 bp downstream of *luxR* start codon (TTCTAA)
T9002-D (C8)	Point mutation	100	-7 site of *luxR* promoter (T-->C)
T9002-D (C9)	Small insertion	100	1-bp insertion 515 bp downstream of *luxR* start codon
T9002-E (C1, C6, C7, C9)	Deletion	50	Deletion that removes B0012 and R0062 (*luxR* promoter)
T9002-E (C2)	Point mutation	50	-7 site of *luxR* promoter (T-->C)
T9002-E (C3)	IS5	50	257 bp downstream of *luxR* start codon (TTCTAA)
T9002-E (C4)	Point mutation	50	-11 site of *luxR* promoter (A-->G)
T9002-E (C5)	Small deletion	50	1-bp deletion 213 bp downstream of GFP start codon
T9002-E (C8)	Point mutation	50	-11 site of *luxR* promoter (A-->T)
T9002-F (C1)	Small deletion	200	1-bp deletion 534 bp downstream of *luxR* start codon
T9002-F (C2)	IS5	200	257 bp downstream of *luxR* start codon (TTCTAA)
T9002-F (C3)	Deletion	200	Deletion that removes B0010, B0012, and R0062
T9002-F (C4, C7)	Deletion	200	Deletion that removes B0012 and R0062
T9002-F (C5)	Small deletion	200	1-bp deletion 529 bp downstream of *luxR* start codon
T9002-F (C6)	IS1	200	277 bp downstream of *luxR* start codon (CAATTA)
T9002-F (C8)	IS5	200	410 bp downstream of *luxR* start codon (GCTTAG)
T9002-F (C9)	Point mutation	200	622 bp downstream of *luxR* start codon (G-->T)
R0011+E0240 (C1-C9)	Deletion	50	Deletion that removes *lacO* and entire -35 promoter sequence
R0040+E0240 (C1-C9)	Deletion	100	Deletion that removes *tetO* and entire -35 promoter sequence
J23101+E0240 (C1)	IS5	300	Scar in between RBS and GFP coding sequence (TACTAG)
J23101+E0240 (C2)	Point mutation	300	-31 site of J23101 promoter (C-->T)
J23101+E0240 (C3)	Point mutation	300	-7 site of J23101 promoter (T-->G)
J23101+E0240 (C4)	IS5	300	Scar in between RBS and GFP coding sequence (TACTAG)
J23101+E0240 (C5)	IS2	300	-9 site of J23101 promoter (GTATTT)*****
J23101+E0240 (C6)	IS5	300	-24 site of J23101 promoter (AGCTAG)
J23101+E0240 (C7)	Point mutation	300	-12 site of J23101 promoter (T-->G)
J23101+E0240 (C8)	Deletion	300	Deletion that removes B0032, E0040, and B0010
J23101+E0240 (C9)	Deletion	300	4-bp deletion of last 3 bases in -10 and 1-bp downstream
J23151+E0240 (C5)	IS5	500	-1 site of promoter (TGCTAG)
J23151+E0240 (C6)	Point mutation	500	-7 site of J23151 promoter (T-->G)
J23151+E0240 (C9)	Point mutation	500	-7 site of J23151 promoter (T-->C)
R0010+E0240+kanR polycis (C2)	IS5	200	Scar in between promoter and RBS (TACTAG)
R0010+E0240+kanR polycis (C3)	IS2	200	-9 site of promoter (CGTATT)*****
R0010+E0240+kanR polycis (C4)	IS1	200	-33 site of promoter (AGGCTT)
R0010+E0240+kanR polycis (C7)	IS2	200	-23 site of promoter (CTTTAT)
R0010+E0240+kanR fusion (C1)	IS5	200	Scar in between RBS and GFP coding sequence (TACTAG)
R0010+E0240+kanR fusion (C2)	IS5	200	Scar in between RBS and GFP coding sequence (TACTAG)
R0010+E0240+kanR fusion (C3)	IS1	200	-11 site of promoter (GCTCGT)
R0010+E0240+kanR fusion (C4)	IS5	200	Scar in between RBS and GFP coding sequence (TACTAG)
R0010+E0240+kanR fusion (C6)	IS5	200	Scar in between promoter and RBS (TACTAG)
R0010+E0240+kanR fusion (C7)	IS2	200	-44 site of promoter in CAP binding site (TTAGGT)*****
R0010+E0240+kanR fusion (C8)	Deletion	200	7-bp deletion that removes entire -35 and one bp upstream
R0010+E0240+kanR fusion (C9)	IS2	200	+5 site of promoter in *lacI* binding site (GGAATT)
R0011+E0240 (C1-C9, +IPTG)	Deletion	500	Deletion that removes *lacO* and entire -35 promoter sequence
R0010+E0240 (C7, +IPTG)	IS5	500	Scar in between promoter and RBS (TACTAG)

* Hybrid B0010 terminator that has the following sequence:
CCAGGCATCAAATAAAACGAAAGGCCCAGTCTTTCGACTGAGCCTTTCGTTTTATTTGATGCCTGG

** Removes part of B0010, all of B0012, R0062 (luxR promoter), and part of E0040 (GFP)

*** Hybrid B0012 terminator that has the following sequence: CCACACTGGCCCACCCGAAGGTGAGCCAGTGTGAC

**** Different hybrid B0012 from the C3 and C8 clones that has the following sequence: CCAGTGTGAC
***** 1-bp insertion of 'T' at target site

Supplementary Table 1 – Genetic circuit mutations in all evolved populations.

For all replicate evolved populations in every circuit, the mutation is described with the timepoint the plasmid was sequenced. In the circuit column, the "C" stands for Clone and the number is the population number (1-9). In clones with IS element mutations, the 6-bp target site upstream of the insertion is given in parentheses in the description.

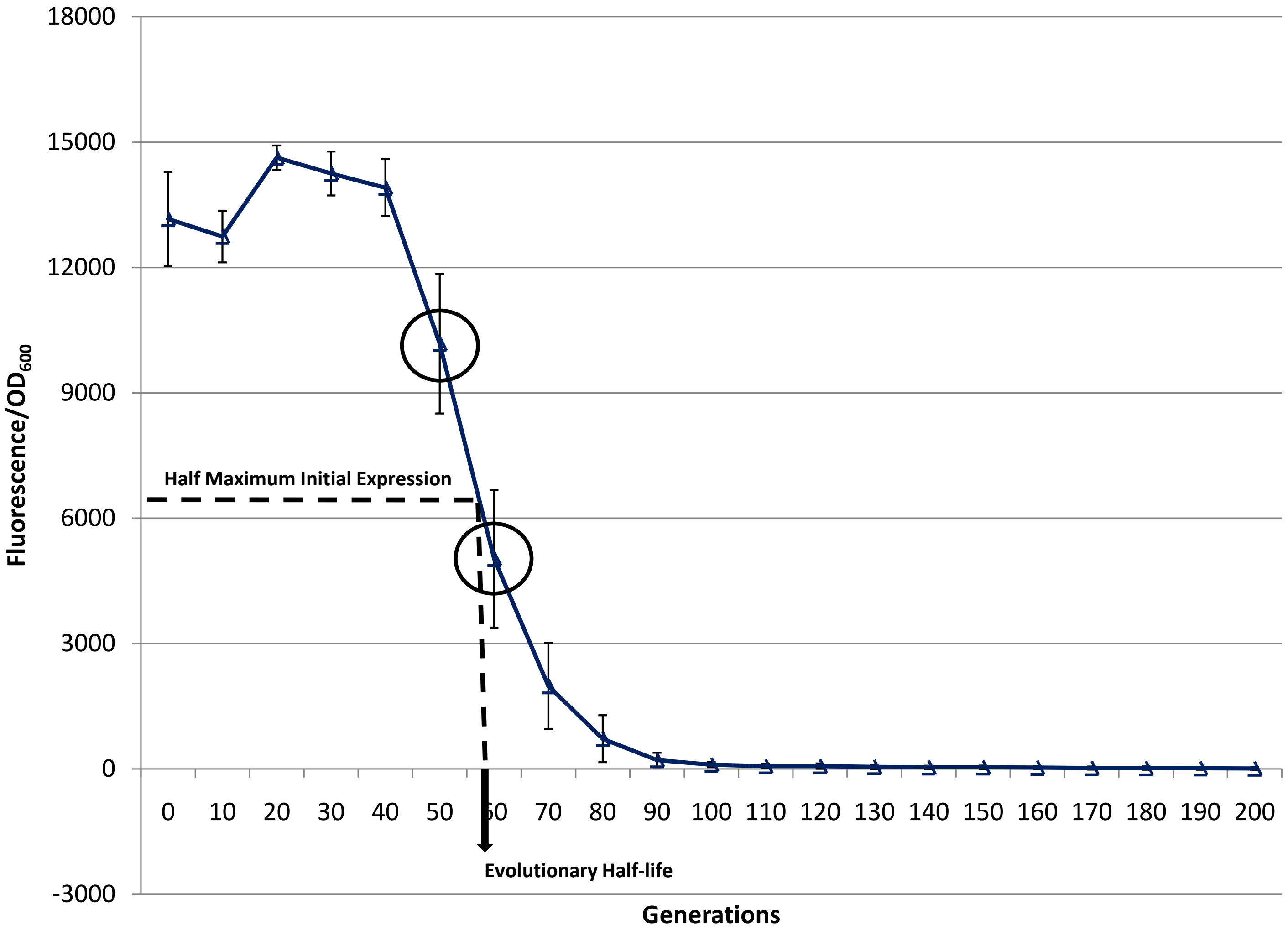

Supplementary Figure 1 - Schematic for evolutionary half-life calculations.
Evolutionary half-life was calculated for each independently evolved population. First, the slope and y-intercept were calculated using the two data points on either side of the half maximum expression value on the evolutionary stability plot (circled). A linear regression on those two data points was performed using the equation y = ax + b, where y = the half maximum initial expression (shown by the dotted black line), a = the slope of the two data points, b = the y-intercept of the two data points, and solving for x gives the evolutionary half-life (shown by the downward pointing arrow).

Evolutionary half-life measurements of individual populations

To determine if there is a relationship between initial expression level of a circuit and its evolutionary half-life, we performed a regression on all evolutionary half-life measurements for every replicate population in the evolved populations from Figure 7 (Supplementary Figures 2 and 3) and the circuits shown in Figures 3-6 (Supplementary Figure 4-8). See the Methods section and Supplementary Figure 1 for details on how evolutionary half-life was calculated.

Supplementary Figures 2 and 3 show the individual data points for evolutionary half-life vs. initial expression level and an exponential fit is shown since the r^2 values are higher than a linear fit. The curves follow a similar trend as those shown in Figure 7. The r^2 values are 0.728 for T9002, 0.875 for T9002-E, 0.707 for R0011+E0240, and 0.609 for R0010+E0240. The intercept, slope, and model p-values of these regressions are all highly significant ($p < 0.0001$), indicating that the correlation between expression and evolutionary half-life for all these circuits is not a coincidence. For these four circuits, the regressions indicate that between roughly 60-90% of the variation in evolutionary half-life can be explained by the initial expression level.

Supplementary Figure 4 shows the individual data points for evolutionary half-life vs. initial expression for all circuits shown in Figures 3-6. There is a rough relationship between expression level and evolutionary half-life where the r^2 value is 0.597 using an exponential fit. The intercept, slope, and model p-values of the regression are all highly significant ($p < 0.0001$). Although initial expression levels may be a rough predictor of evolutionary stability, mutation rate and fitness calculations will likely help improve this prediction.

Evolutionary half-life measurements vs. initial expression levels were also plotted individually for the T9002, promoter library, KanR, and LacI-inducible circuits (Supplementary Figures 5-8). The T9002 circuits show the best r^2 fit (0.86), followed by the LacI-inducible circuits (0.691), KanR circuits (0.482), and finally the promoter library circuits (0.403). It is unclear why promoter strength does not correlate well with evolutionary half-life despite the regression values being highly significant ($p < 0.0001$), but it may be because these promoters have very different mutation rates or mutations in the chromosome are selected for. Besides R0011 and R0040 that have repeated operator sequences, there are no other obvious reasons why the other promoters should have different mutation rates. The J23101+E0240 circuit had mutations in all nine replicate evolved populations, whereas there were none in some other populations, indicating that mutations in the chromosome may have been selected for instead. The other possibility for why promoter strength does not correlate well with evolutionary half-life is that since these promoters are not regulated, there may be more variability in their expression over time due to the lack of transcription factors that can more tightly control expression. On the other hand, the T9002 circuits evolutionary half-life measurements correlate well with initial expression level. In fact, if the mean evolutionary half-life measurements (Table 1) are plotted against the mean initial expression level, the r^2 value is 0.98. This high correlation may be because in these T9002 circuits, expression is tuned using different transcriptional terminators that does not change noisy transcription levels or promoter mutation rate and is more dependent upon initial expression levels alone.

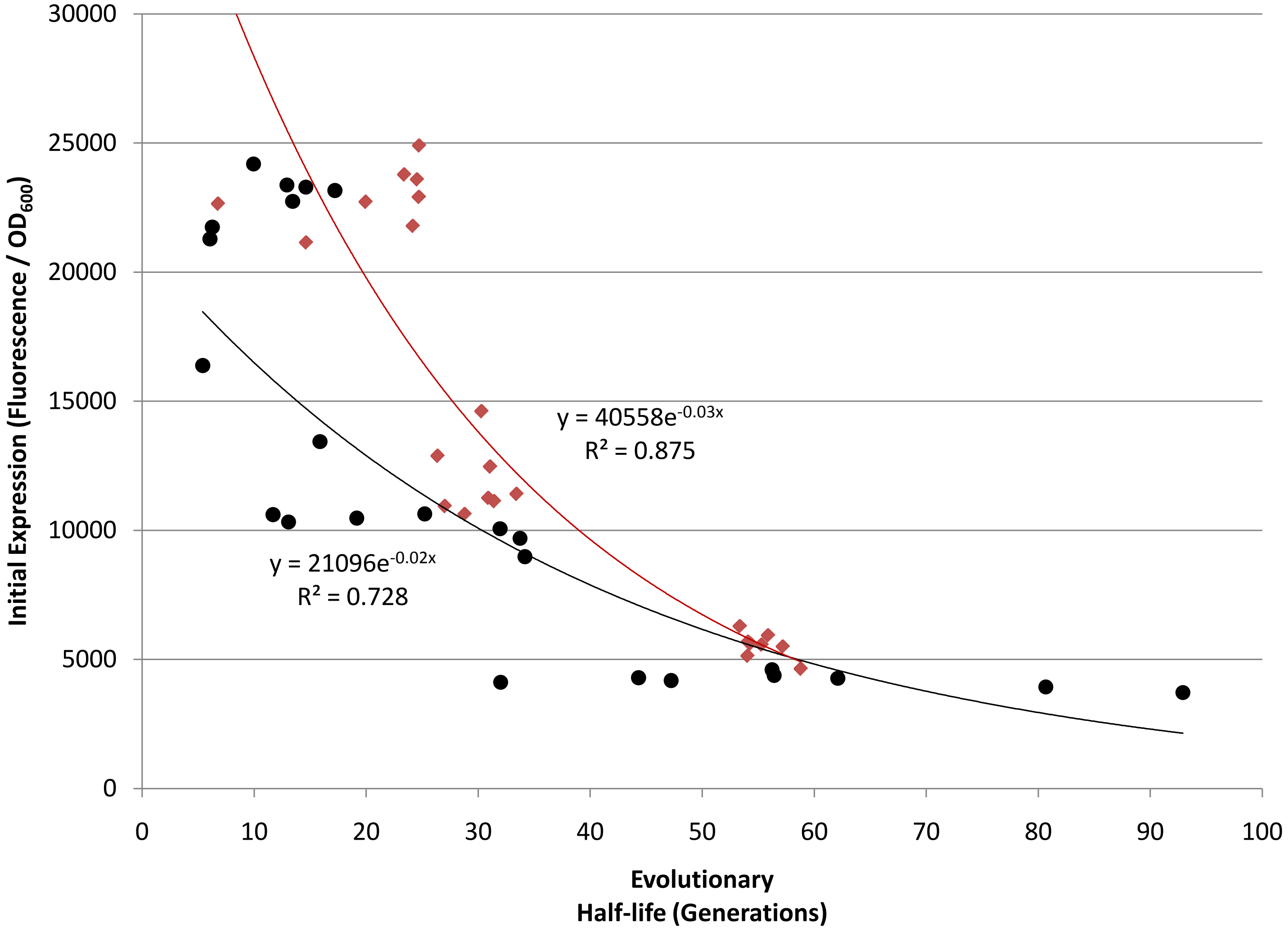

Supplementary Figure 2 - Regression of evolutionary half-life vs. initial expression for T9002 and T9002-E evolved populations with different AHL concentrations (Figure 7a).

Individual data points of evolutionary half-life vs. initial expression are plotted for each replicate evolved population in T9002 (black circles) and T9002-E (red diamonds). An exponential fit is shown by the black or red lines along with the regression equations and r^2 measurements.

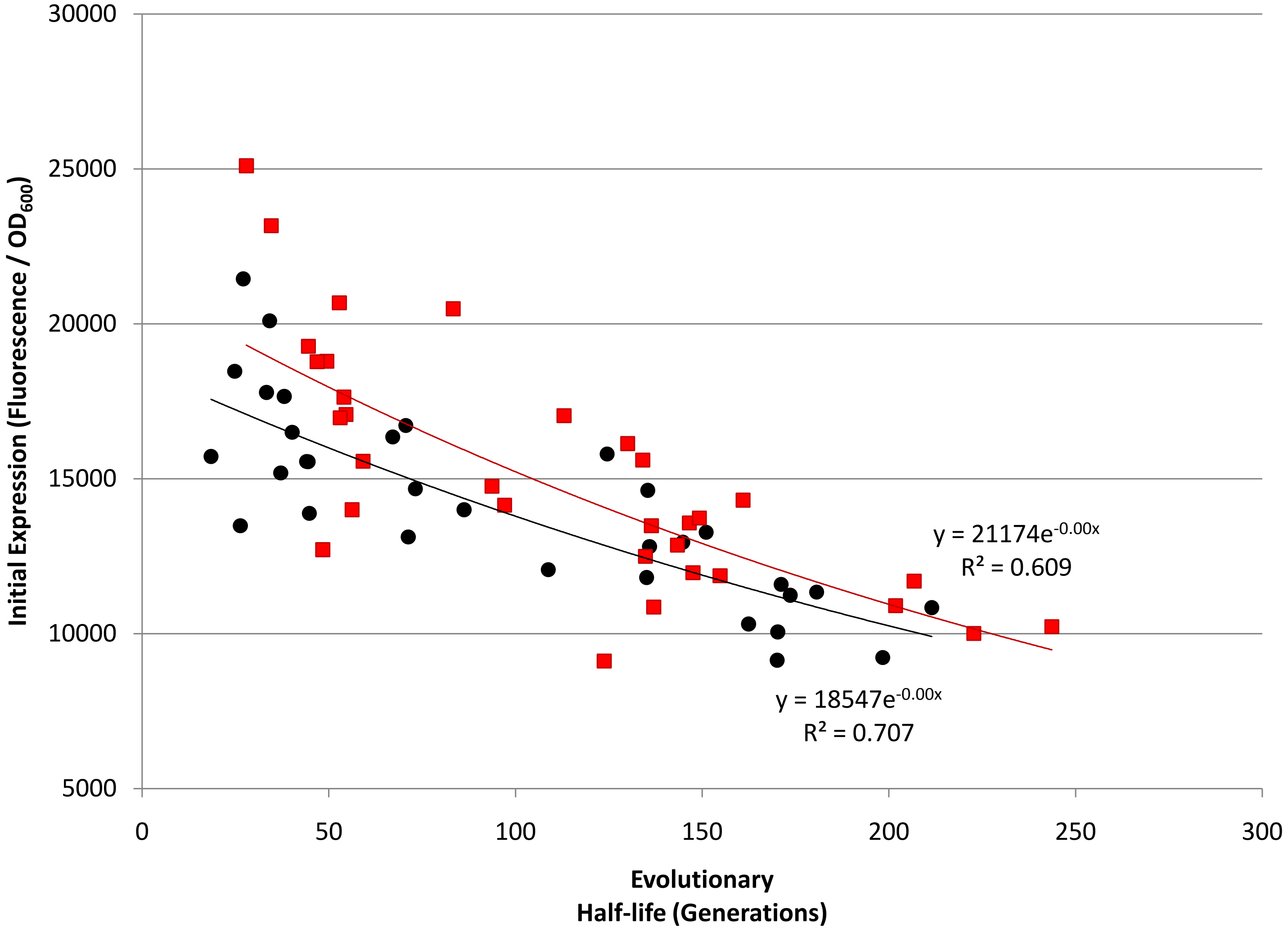

Supplementary Figure 3 - Regression of evolutionary half-life vs. initial expression for R0011+E0240 and R0010+E0240 evolved populations with different IPTG concentrations (Figure 7b).
Individual data points of evolutionary half-life vs. initial expression are plotted for each replicate evolved population in R0011+E0240 (black circles) and R0010+E0240 (red squares). An exponential fit is shown by the black or red lines along with the regression equations and r^2 measurements.

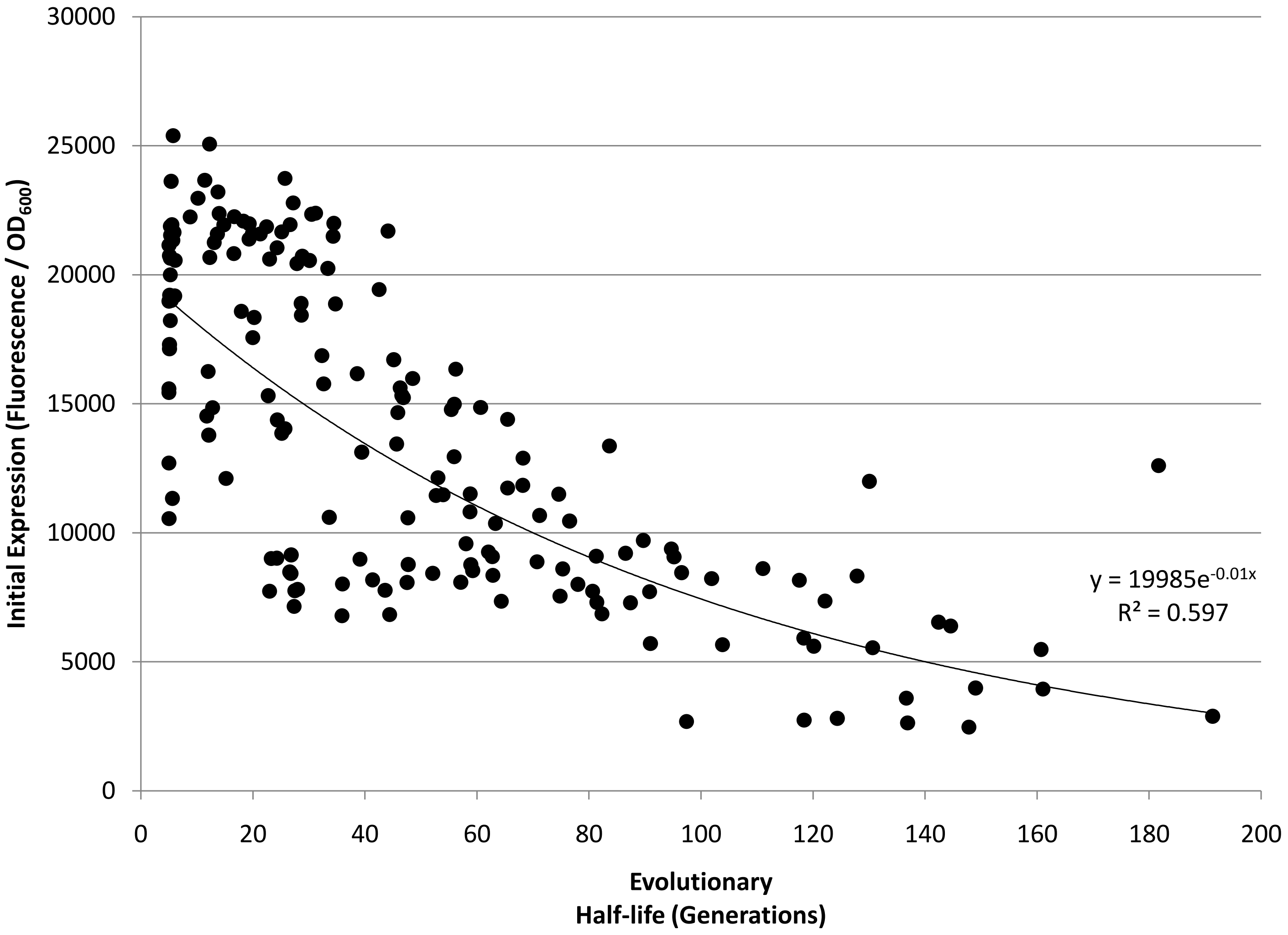

Supplementary Figure 4 - Regression of evolutionary half-life vs. initial expression for all circuits. Individual data points of evolutionary half-life vs. initial expression are plotted for each replicate evolved population in all circuits. An exponential fit is shown by the black line along with the regression equation and r^2 measurement.

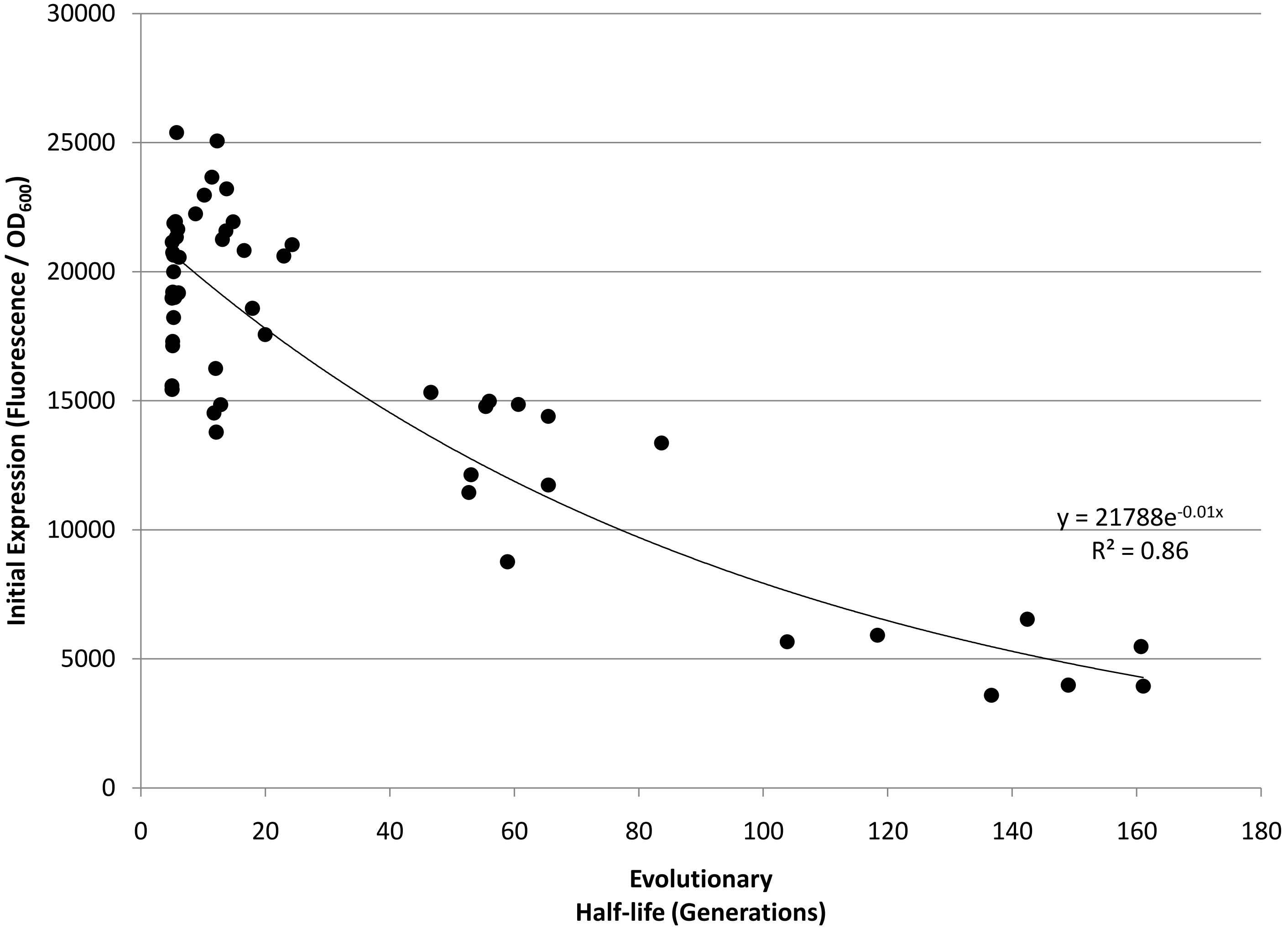

Supplementary Figure 5 - Regression of evolutionary half-life vs. initial expression for T9002 circuits. Individual data points of evolutionary half-life vs. initial expression are plotted for each replicate evolved population in all T9002 circuits. An exponential fit is shown by the black line along with the regression equation and r^2 measurement.

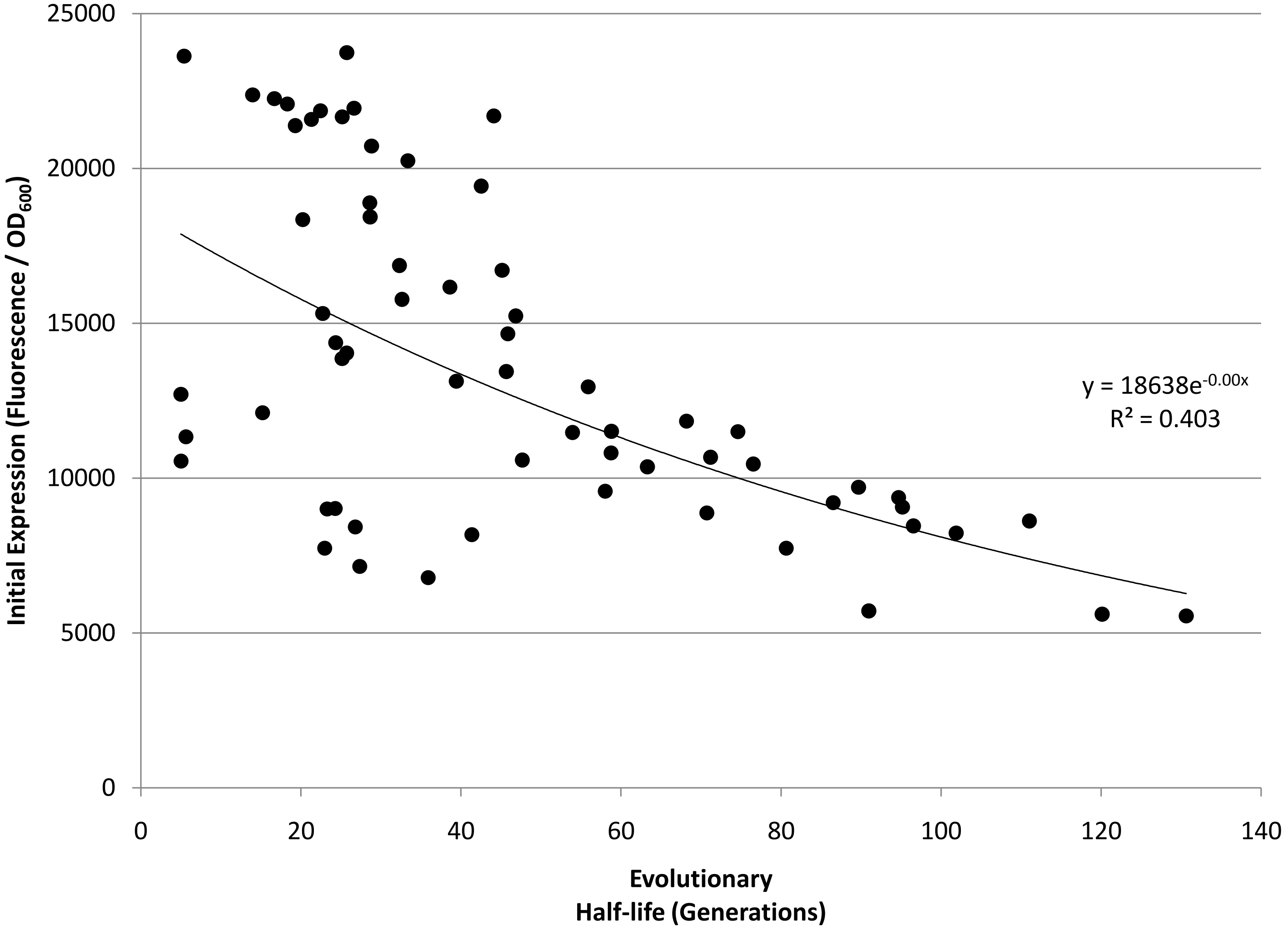

Supplementary Figure 6 - Regression of evolutionary half-life vs. initial expression for promoter library circuits.
Individual data points of evolutionary half-life vs. initial expression are plotted for each replicate evolved population in all promoter library circuits. An exponential fit is shown by the black line along with the regression equation and r^2 measurement.

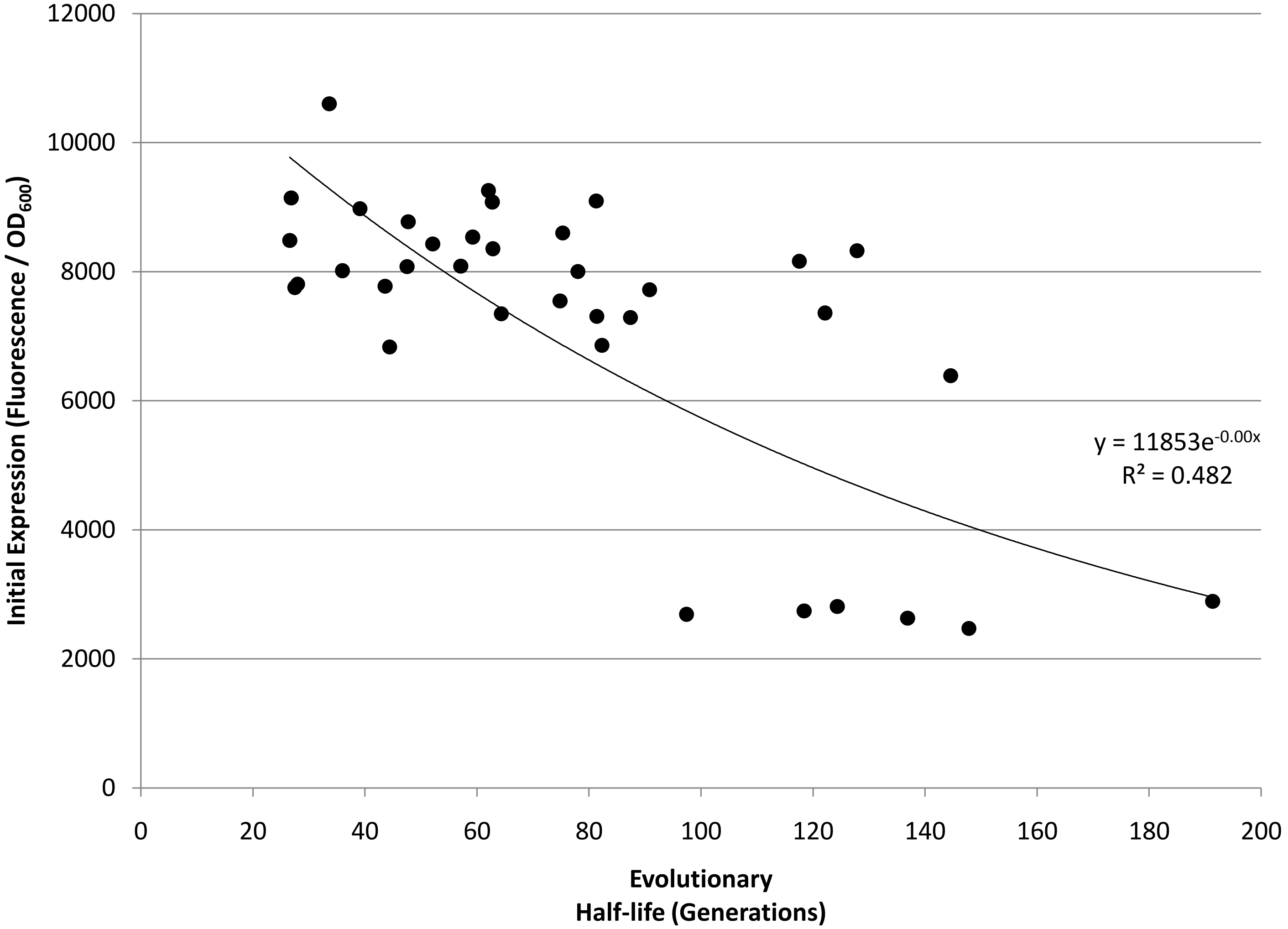

Supplementary Figure 7 - Regression of evolutionary half-life vs. initial expression for KanR circuits. Individual data points of evolutionary half-life vs. initial expression are plotted for each replicate evolved population in all KanR circuits. An exponential fit is shown by the black line along with the regression equation and r^2 measurement.

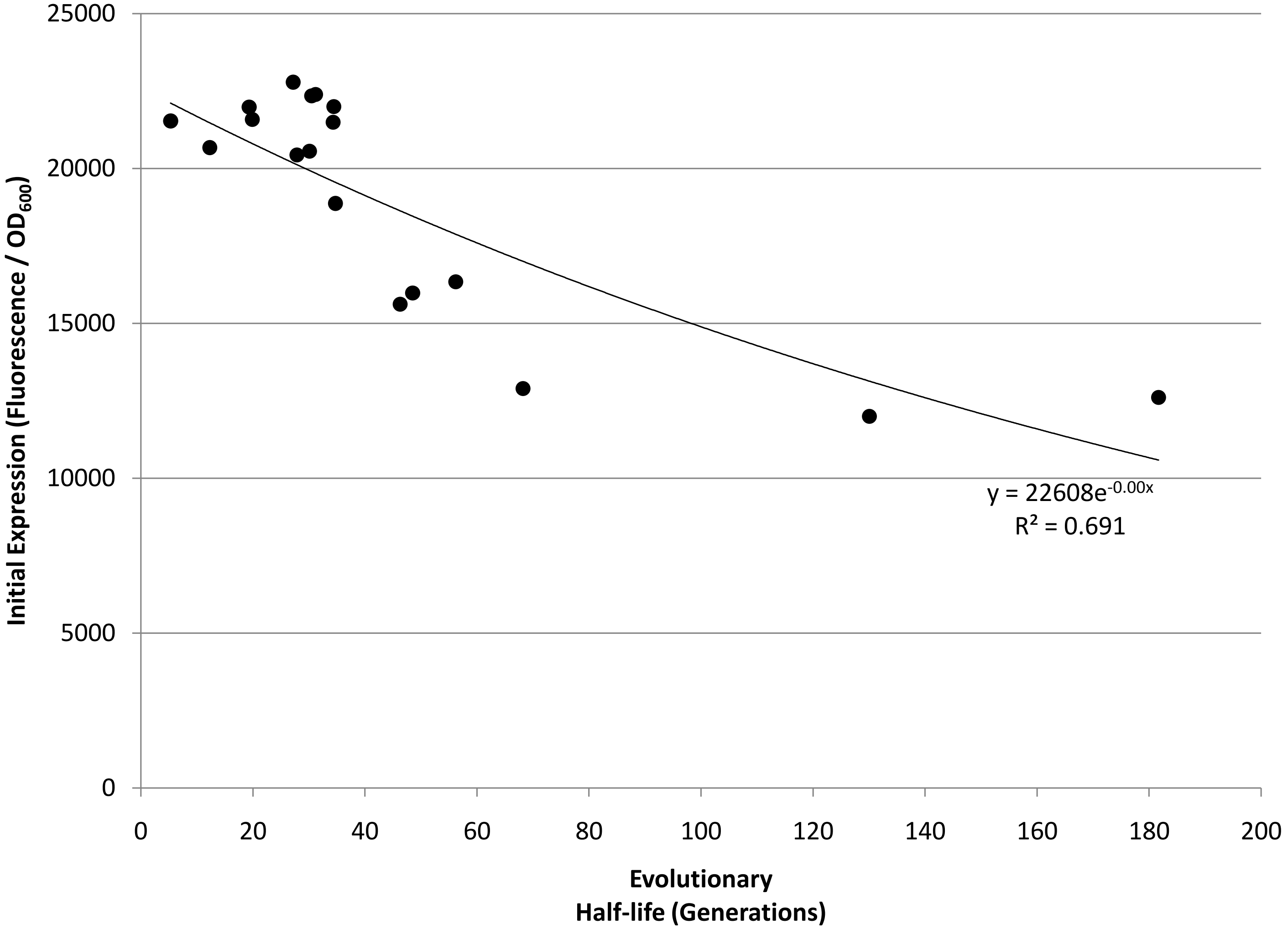

Supplementary Figure 8 - Regression of evolutionary half-life vs. initial expression for LacI-regulated circuits.

Individual data points of evolutionary half-life vs. initial expression are plotted for each replicate evolved population in all LacI-regulated circuits. An exponential fit is shown by the black line along with the regression equation and r^2 measurement.

Testing for mutations on the chromosome in evolved strains

We found that certain circuits did not have mutations after propagated a certain number of generations even though the expression level of the population decreased over time. The only explanation for this is that there are other mutations on the origin of replication on the plasmid or mutations on the chromosome. Chromosomal mutations may somehow decrease expression of the circuit either by decreasing plasmid copy number or through another mechanism. We sequenced plasmids from one clone in every circuit and did not find any origin of replication mutations in evolved plasmids vs. their progenitors. In fact, all plasmids with mutations were transformed back into the progenitor strain to ensure that loss-of-function was due to mutations on the plasmid and this was indeed the case. To test whether there were mutations in the chromosome that are not on the plasmid, we performed additional experiments on two evolved strains, R0010+E0240 and R0010+E0240 *kanR* polycistronic propagated with kan in the media. If the original plasmid is transformed back into the evolved strain cured of its plasmid, then expression should be low relative to the progenitor strain if there are mutations that lower expression on the chromosome.

First, we cured the plasmids from the R0010+E0240 and R0010+E0240 *kanR* polycistronic (+kan) evolved strains (generation 280 and 500, respectively). We then made the progenitor and evolved strains competent for transformation and transformed the progenitor and evolved plasmids back into these strains (Supplementary Figure 9). The expression level was measured for each of the four progenitor/evolved strain/plasmid combinations (Supplementary Figure 10). This figure shows that the evolved strains have very low expression when either the progenitor and evolved plasmids are transformed. In contrast, both progenitor and evolved plasmids have high expression in the progenitor strains. This indicates that the expression differences are due to unknown mutations in the chromosome and are not in the plasmids.

As a final experiment, we also extracted the plasmids from the eight strains shown in Supplementary Figure 10. For the R0010+E0240 strains, the progenitor strains (with the progenitor and evolved plasmids) had roughly 3-fold more plasmid DNA compared to the evolved strains (with the progenitor and evolved plasmids). These results are repeatable and plasmids were run out on a gel to confirm the difference in plasmid DNA quantity. This suggests that an unknown mutation(s) on the chromosome decreases copy number in the evolved R0010+E0240 strain. On the other hand, we found no significant difference in the amount of plasmid DNA in the R0010+E0240 *kanR* polycistronic (+kan) strains. We also sequenced the origin of replication in three clones in both KanR circuits propagated with and without kan and found no mutations. Therefore, the expression level decreases over time by some unknown mechanism due to an unknown mutation(s) on the chromosome.

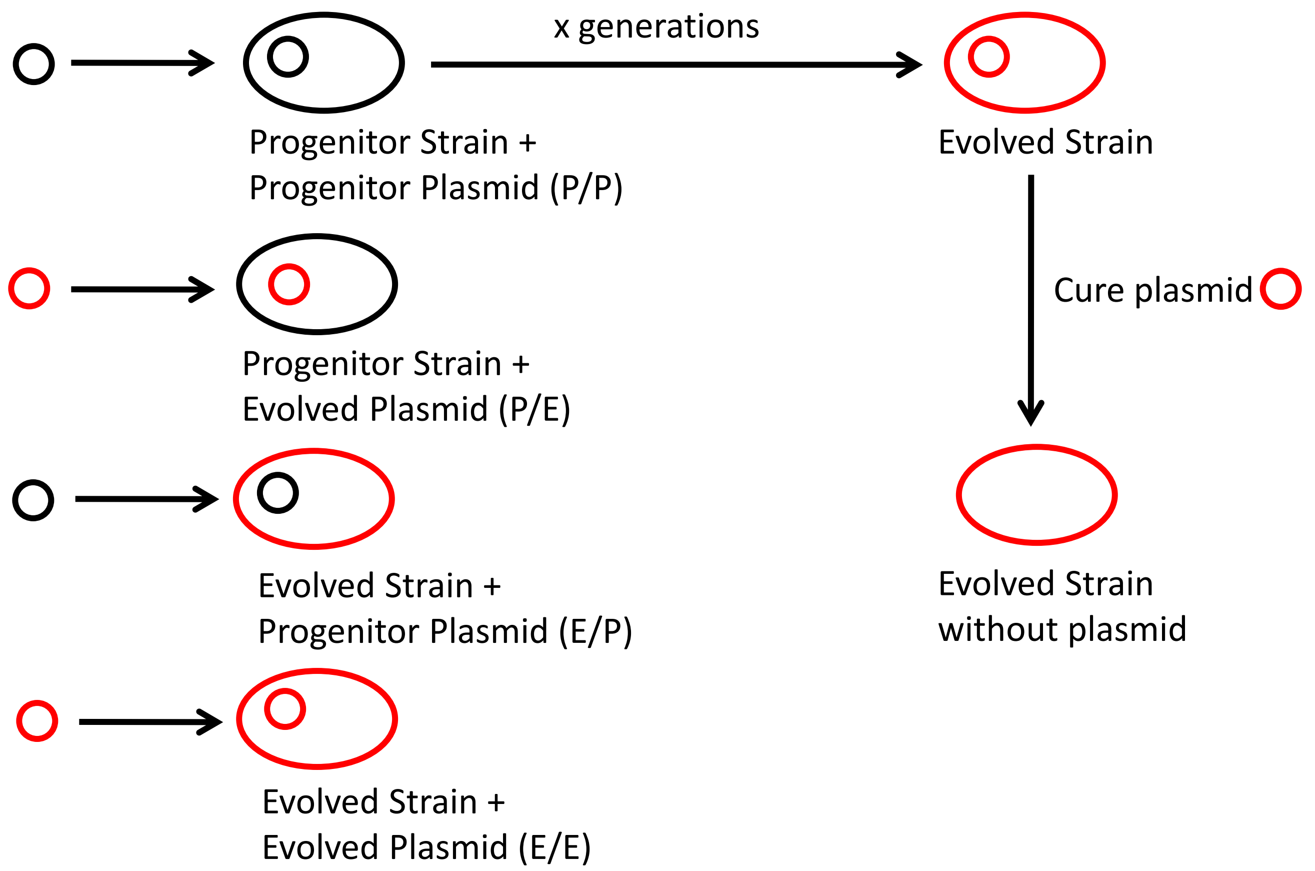

Supplementary Figure 9 – Schematic of methods for progenitor and evolved plasmids transformed into the progenitor and evolved strains.
The progenitor (black) strain and plasmid (P/P) are propagated over multiple generations to the evolved (red) strain and plasmid. The evolved plasmid is cured from the evolved strain (see Methods for details). The progenitor plasmid can then be transformed into the evolved strain (E/P). Likewise, the evolved plasmid can be transformed into the progenitor strain (P/E) and re-transformed into the evolved strain (E/E).

A.

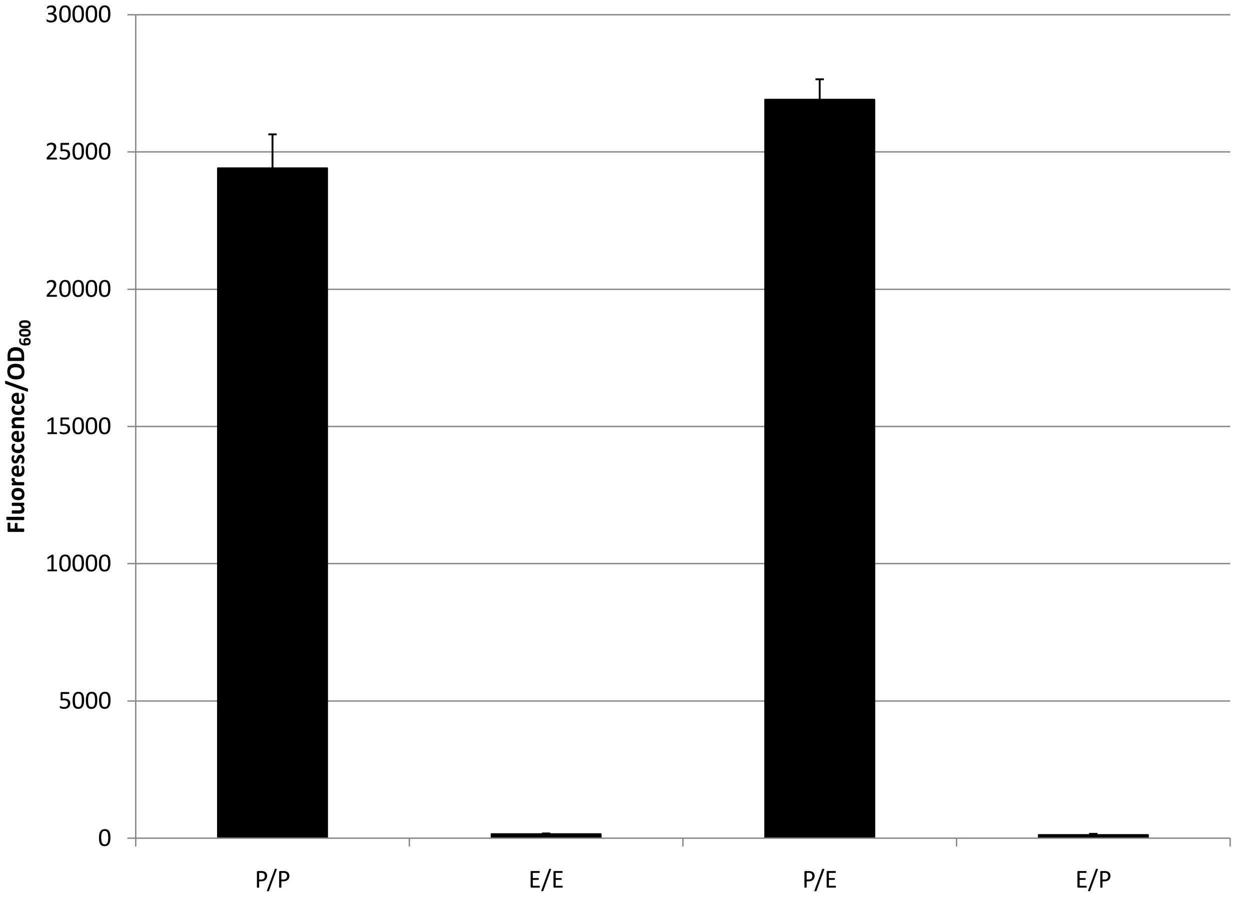

30000
25000
20000
15000
10000
5000
0
Fluorescence/OD600
P/P
E/E
P/E
E/P

B.

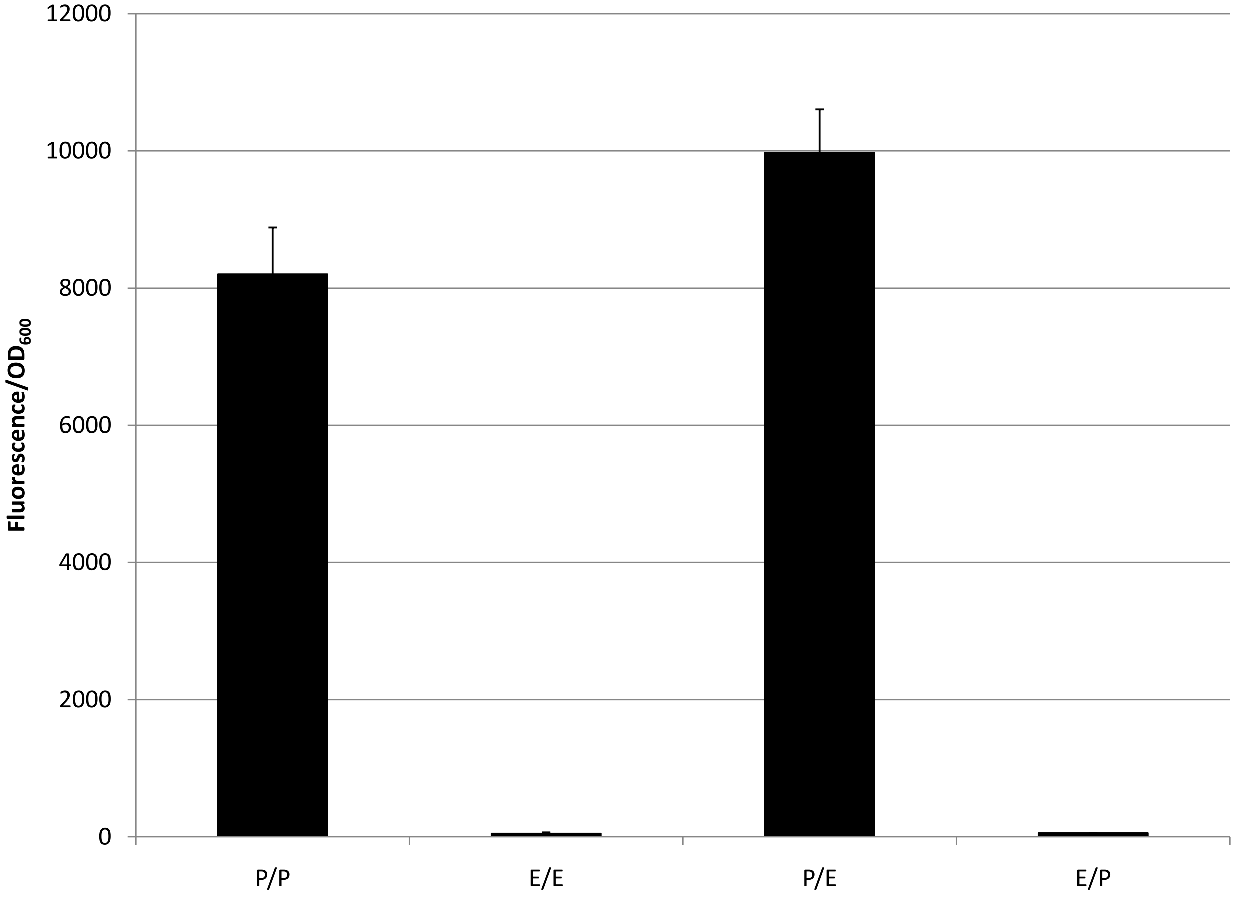

12000
10000
8000
6000
4000
2000
0
Fluorescence/OD600
P/P
E/E
P/E
E/P

Supplementary Figure 10 – Expression levels of the progenitor and evolved plasmids transformed into the progenitor and evolved strains.

(A) The expression level is shown for each of the four strain/plasmid combinations in the R0010+E0240 circuit. The evolved strain and plasmid is from the 280 generations timepoint. The four combinations are: P/P (progenitor strain with progenitor plasmid), E/E (evolved strain with evolved plasmid), P/E (progenitor strain with evolved plasmid), and E/P (evolved strain with progenitor plasmid). Error bars represent one standard deviation from the mean. (B) The expression level is shown for each of the four strain/plasmid combinations in the R0010+E0240 *kanR* polycistronic circuit. The evolved strain and plasmid is from the 500 generations timepoint. The four combinations are: P/P (progenitor strain with progenitor plasmid), E/E (evolved strain with evolved plasmid), P/E (progenitor strain with evolved plasmid), and E/P (evolved strain with progenitor plasmid). Error bars represent one standard deviation of nine replicates.
